# A new high-accuracy QSAR model based on ultra-curated data for predicting thyroid receptor-related endocrine activity

**DOI:** 10.3389/ftox.2026.1852502

**Published:** 2026-08-20

**Authors:** Emel Ay-Albrecht, Zlatomir Todorov, Franklin J. Bauer, Marie Darracq-Ghitalla-Ciock, Carole Charmeau-Genevois, Satinder S. Sarang, Paul C. Thomas

**Affiliations:** 1 KREATiS SAS, L’Isle d’Abeau, France; 2 Product Stewardship, Shell, Houston, TX, United States

**Keywords:** Applicability domain (AD), chemical toxicity, data curation, endocrine disruption, *in silico* modelling, machine learning, qsar modelling, thyroid receptors alpha and beta

## Abstract

**Introduction:**

Endocrine active substances interfere with hormone signalling and represent a major regulatory concern. The thyroid system is particularly vulnerable, with disruption linked to neurodevelopmental, metabolic, and cardiovascular effects. Current in vitro assays may improve mechanistic understanding but are resource-intensive while screening databases (e.g., Tox21 assays) is insufficient for large-scale chemical coverage or for assessing new materials. In silico tools such as QSAR models offer scalable alternatives, yet existing thyroid receptor (TR) models suffer from poor data curation, class imbalance, and limited regulatory acceptance.

**Methods:**

This study aimed to develop a high-accuracy QSAR model, aligned with the OECD QSAR Assessment Framework, to accurately predict TR-related endocrine activity, thereby supporting the reduction of animal testing and regulatory decision-making. In this work, data from Tox21 TR luciferase agonist and antagonist assays were combined with curated BindingDB data, followed by expert review of activity-cytotoxicity relationships and strict filtering of low-affinity interactions (termed here as “ultra-curation”). Agonists and antagonists were grouped together as “actives”. The final dataset comprised 291 active and 6,049 inactive compounds. Each substance was associated with a standardised SMILES, to locate duplicates (subsequently removed), and the dataset split into training, internal validation, and external validation sets with chemical diversity balancing. A Support Vector Machine (SVM) classifier using circular fingerprints (FCFP6) was optimised via grid search. Model performance was evaluated with cross-validation and external validation, complemented by an applicability domain (AD) framework based on analogues.

**Results:**

The SVM achieved strong performance in training (balanced accuracy = 96.03%; AUC-ROC = 99.91%; AUC-PR = 99.21%) and robust performance on the external validation set (balanced accuracy = 92.46%; sensitivity = 87.65%; specificity = 99.42%). Incorporation of the AD framework improved minority class (active) detection, raising balanced accuracy to 96.15%, sensitivity to 92.30%, and specificity to 100%, while excluding ~14% of compounds with uncertain predictions.

**Discussion:**

The QSAR model developed here demonstrates good predictive accuracy for thyroid receptor-related endocrine activity and addresses key regulatory concerns through rigorous data ultra-curation and applicability domain assessment. This high-accuracy QSAR provides a promising in silico tool to support integrated approaches to testing and assessment (IATA) and regulatory evaluation of endocrine disruption potential.

## 1 Introduction

In 2002, the World Health Organisation (WHO) defined an Endocrine Disruptor as “a substance or mixture that alters function(s) of the endocrine system and consequently causes adverse health effects in an intact organism, or its progeny, or (sub) population” (WHO and IPCS, 2002). Based on this definition, ECHA/EFSA defines in 2018 when a substance is considered as having an endocrine disrupting mode of action, *i.e*., there is a biologically plausible link between the adverse effect and the endocrine activity (ECHA/EFSA, 2018). The endocrine modulation can occur at multiple levels, including hormone synthesis, transport, receptor binding, and metabolism (La Merrill et al., 2020). A striking example is diethylstilbestrol, a synthetic non-steroidal oestrogen once prescribed for miscarriage prevention, which was later shown to induce foetal malformations through oestrogen receptor activation (Robotti, 2021). In addition to receptor-mediated effects, diethylstilbestrol also induces epigenetic modifications, leading to transgenerational outcomes such as carcinogenicity and teratogenicity (Klip et al., 2002). This illustrates the diversity of endocrine-disrupting mechanisms and has fuelled scientific and regulatory debate regarding their identification and classification (Autrup et al., 2020; Brescia, 2020). Regulatory agencies and authorities, however, have emphasised receptor-mediated mechanisms as a primary basis for classification. In 2023, new hazard classes for endocrine disruptors have been introduced in Classification, Labelling and Packaging (CLP) Regulation. In 2024 the European Chemicals Agency (ECHA) incorporated endocrine disruption potential into its CLP guidelines, further reinforcing the need for reliable methods to identify such chemicals (ECHA, 2024). In response, a variety of *in vitro* assays have been developed to detect agonist or antagonist activity, including hormonal pathways of high concern such as the thyroid axis (OECD, 2024a).

Currently, no new GHS hazard class for ED is available. In 2025, the OECD *ad hoc* group of various experts reviewed existing GHS hazard classes (*e.g.,* carcinogenicity, reproductive toxicity, specific target organ toxicity, hazardous to the aquatic environment) that may include endocrine disrupters. The group agreed that there is a gap in GHS hazard classes regarding the possibility to identify EDs as such, according to the WHO/IPCS definition. In particular, inclusion of mechanistic data is not mentioned as part of the identification of most hazards (mutagenicity is an exception), which would be a gap in the current ability to identify a chemical as an ED under the GHS (OECD, 2025a). Moreover, using the IPCS/WHO definition of EDs, no stand-alone method can be used to identify a substance as an ED, and rather, methods must be used in combination. In this regard, *in silico* methods such as those described here, have their place in tier one alongside other studies in the battery to be implemented to identify EDs chemicals (ECHA/EFSA, 2018).

The thyroid gland produces the hormones triiodothyronine (T_3_) and thyroxine (T_4_), which regulate numerous physiological processes across multiple organ systems. Thyroid hormones control metabolic functions through effects on the liver, including regulation of thermogenesis, glucose production and uptake, cholesterol homeostasis, and fatty acid oxidation. They also modulate cardiovascular function by influencing heart rate and contractility, and contribute to musculoskeletal regulation via bone cell differentiation, muscle contraction, and creatinine metabolism. Most critically, thyroid hormones are indispensable for brain development, where they direct neuronal maturation, axonal myelination, and synaptic connectivity (Norman and Henry, 2015).

Consequently, disruption of thyroid signalling can have profound and lasting consequences, particularly during foetal and neonatal development (Feldt-Rasmussen and Mathiesen, 2011; UNEP/WHO, 2012). While hyper- and hypothyroidism are commonly managed as clinical conditions arising from autoimmune diseases (*e.g*., Hashimoto’s disease, Graves’ disease) or iodine imbalance, growing evidence implicates exposure to environmental chemicals as a cause of thyroid disruption (Fernandez et al., 2018; Taha et al., 2020; Marques et al., 2022). For example, certain benzophenones have been associated with thyroid-related effects, although their precise mechanisms of action remain poorly defined (Axelstad et al., 2013; Zhang et al., 2018; Mustieles et al., 2023) and may not be related to an interaction with the thyroid hormone receptors.

Historically, thyroid disruption was assessed through reproductive and repeated-dose studies in rodents (OECD, 2025b). However, in line with the 3R principles (Replacement, Reduction, Refinement), mechanistic *in vitro* approaches have gained importance. In 2024, the OECD reviewed eleven *in vitro* methods for thyroid disruption assessment, and large-scale screening programs such as the U.S. Tox21 initiative employed assays like the GH3 cell-based qHTS luciferase system (OECD, 2024a). While these assays improve mechanistic understanding and reduce animal use, they remain resource-intensive and insufficient to screen the vast number of chemicals in commerce or predict the activity of new ones. Moreover, they cannot fully replace *in vivo* testing.

To provide upstream strategic direction to experimental strategies, *in silico* approaches such as quantitative structure-activity relationship (QSAR) modelling is increasingly promoted within integrated approaches to testing and assessment (IATA). QSAR models offer the potential for high-throughput, cost-effective prediction of endocrine activity and when built and used appropriately, can indicate and limit future testing only to areas of potential concern. However, most existing models for thyroid receptor-related activity are limited in accuracy, rely on poorly curated datasets, and fall short of the standards required for regulatory use outlined in the OECD QSAR Assessment Framework (Sellami et al., 2022; OECD, 2024b; Vergauwen et al., 2024; Evangelista and Papa, 2025). These shortcomings result in frequent false positives and undermine regulatory acceptance.

In this context, the aim of this work was to develop a new high-accuracy QSAR model, further to a call for proposals in the NC3Rs “CRACK-IT Challenge” research program (NC3Rs, 2026), specifically designed to predict thyroid receptor-related endocrine activity. The model described here integrates curated datasets by toxicologists, offering improved predictive performance and enhanced regulatory applicability.

## 2 Materials and methods

### 2.1 Dataset construction and analysis

#### 2.1.1 Dataset sources

The main source of data used to train and validate the model was the set of multi-concentration activity curves resulting from the GH3 Cell-Based qHTS luciferase assay to identify agonist or antagonist of the TR signalling pathway (Freitas et al., 2014). The dataset was primarily sourced from public invitroDB v3.3 (Williams et al., 2017; EPA’s National Center for Computational Toxicology, 2020).

(named “Tox21” in the following sections), and the following assays were used to provide data for this model.

- Tox21_TR_LUC_GH3_Agonist
- Tox21_TR_LUC_GH3_Antagonist
- Tox21_TR_LUC_GH3_Agonist
- Tox21_TR_LUC_GH3_Antagonist_viability

Since this dataset was found to contain only a small proportion of ‘actives’ (*i.e.*, agonists or antagonists), it was complemented by the addition of positive *in vitro* data from the BindingDB database (Liu et al., 2025), where data come from manual curation of articles in selected scientific journals and US patents by the BindingDB team (named “BindingDB” in the following sections). Quantitative values of the activity and/or the affinity from *in vitro* experiments involving compounds and target proteins were extracted. The data in BindingDB do not necessarily come from the same assay type as those in the Tox21 dataset but data selected from this database are based on similar cell-based reporter assays providing EC50 values or IC50 values against a reference T3 activation of TR in all cases.

#### 2.1.2 Curation method

Available SMILES were standardised by removal of salts, stereochemistry was not considered, and a selection of a single converging tautomeric state was carried out. Hence, using translation to InChiKey from the canonical SMILES of mono-constituents, alternative SMILES codes in the input data representing the same principal compounds were removed, resulting in 8,282 standardised SMILES allowing description of 7,155 mono-constituents and 1,816 multi-constituents (after salt removal). The subsequent input data analysis and model development used only records of mono-constituent substances.

The Tox21 actives were further validated by experts through the comparison of activity curves versus cytotoxicity curves. The rational of this comparison arose from detection of extreme cellular stress, ascertained by a decrease in intracellular protease level and cell membrane integrity (Paul-Friedman et al., 2019). In such cases the specific activity on THRs monitored by the ATP-dependent luciferase becomes irrelevant, since a dying cell can also be measured to have decreased ATP levels (Freitas et al., 2014). Hence, the decision to change the classification label from the previous ‘Active’ to curated ‘Inactive’ in such conditions is supported by the assumption that the monitored receptor transactivation signal depends on healthy cell metabolism.

We empirically calibrated the computational formula for the delay between activity and cytotoxicity signals to align with expert assessment (Supplementary Figure 6). A scalable threshold was established to monitor the overlap of dose-response curves of both endpoints, thereby hinting to contrast genuine antagonism from cytotoxicity artifacts. This distinction is critical, as cell death occurring within the antagonist concentration range can suppress transactivation signals, mimicking specific inhibition (Freitas et al., 2014).

The cytotoxic activity of tested compounds was assessed throughout the Tox21 program by monitoring cell protease levels and cell membrane integrity using CellTiter-Fluor technology in the same wells as the antagonist assay, prior to adding the reagent for luciferase activity measurement (National Center for Biotechnology Information, 2014; Paul-Friedman et al., 2019). However, this measurement is only a partial descriptor of cell viability. It was assumed that any specific activity of the test compounds would be relevant only at concentrations below the cytotoxicity limit observed in the satellite assay (Tox21_TR_LUC_GH3_Antagonist_viability), and it was also hypothesised that the cell could start to be non-viable at concentrations slightly lower than the measured level in the viability assay by monitoring protease levels, thus apparent endocrine activity starting at concentrations below observed cytotoxicity could be symptomatic of the loss of viability rather than a true specific interaction with THRs. An alternative criterion not used in this study was proposed by Freitas et al. (2014), where the authors applied a safety factor to limit the risk that observed antagonist activity is due to cytotoxicity: “[antagonist]IC50 values should be at least three times lower than the IC50 in the viability assay”.

The raw data quality assessment and filtering were applied by US EPA as defined in the database release documentation (EPA’s National Center for Computational Toxicology, 2020). Nevertheless, further curation of the input dataset was semi-automated to reproduce the process applied by human experts manually examining the quality of the Tox-21 assay data for a given tested substance. At this stage, various parameters of the models fitted to the raw signal in the original database were compared between activity assays and satellite viability assays (datapoints from the experimentally collected data were not individually considered). Substances remaining with the class label ‘*Active*’ (positive) after the automated curation were further validated by qualified experts examining the ensemble of experimental datapoints within an assay record. Subsequent manual curation of fitted models, preserving the positivity class label, considered additional external knowledge criteria for known biological or optical activity of substances interfering with the transactivation assay. For instance, specific luciferase inhibitors have been identified (Yonchev and Bajorath, 2020) that would lead to apparent antagonist signals in the assays considered in this article. A small sample of substances which were labelled as “inactive” after the automated curation were also reviewed by experts, confirming that all of them were also interpreted as “inactive” by experts.

In our curation protocol, a fitted model is considered representative of genuinely positive *in vitro* activity if it fulfils the following criteria:

- As a minimum it at least reaches the activity threshold for each endpoint defined by the US EPA in the design of the database. These thresholds were strictly accepted without modification.
- Its shape is: (i) a fully observed ascending sigmoidal curve (*i.e.,* one can see the minimum activity plateau at ca. 0% and an ascending part) (ii) a partially observed sigmoidal curve (*i.e.,* the minimum cannot be seen as activity appears to start below the lowest tested concentration); that can even appear as a plateau when the maximum activity was reached below the lowest tested concentration; (iii) a fully observed ‘gain-loss’ curve; (iv) a partially observed ‘gain-loss’ curve that appears to start with a plateau already reached before the lowest tested concentration and beginning to decrease after the third lowest experimentally tested concentration. “Gain-loss” models partially represented over the tested concentration range and exhibiting only the descending sigmoid section from the lowest tested concentration were not accepted as “Active”, because these fitted characteristics easily arise from optimal curve-fitting of noisy data.
- In the specific case of ‘gain-loss’ models the plateau must be represented by more than one tested concentration above the activity threshold.

**FIGURE 1:**
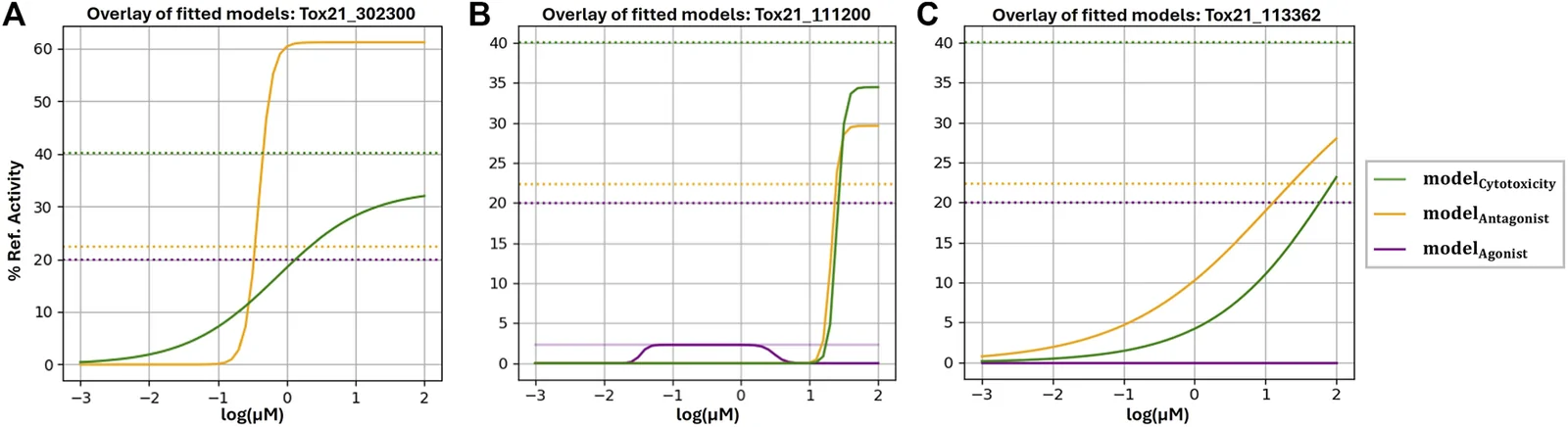
Comparison of activity and cytotoxicity curves from Tox21. **(A)** Viability and antagonist curves have a similar activity-concentration profile. **(B)** The viability assay gives a clear dose-response but less strong than the antagonist assay, not reaching the viability activity cut-off = 40%. **(C)** The viability and antagonist curves have similarly strong responses, but the viability curve crosses the background noise level at a concentration ca. 10 times higher (+1 log unit) than the antagonist curve.

If activity did not start at a significantly lower concentration than cytotoxicity, it was reassigned as “*not active up to the cytotoxicity limit*” or more simply “*inactive*”. Confirmed agonists or antagonists below the cytotoxicity limit were labelled as “*active*”. Some examples of such situations that were interpreted as “*not active up to the cytotoxicity limit*” are shown in Figure 1. The calculation of the “delay” between apparent activity and cytotoxicity and the defined threshold to consider that the curves are sufficiently close to each other is described in the supplementary information.

Subsequently, substances for which the activity raised above noise level only at the highest concentration tested (between 50 and 100 µM depending on the samples) were also not accepted as positives, since cytotoxicity had a high chance of causing this apparent activity at such high concentrations (Supplementary Table S7: ∼30% of cytotoxicity labels assigned to substances at concentrations <15µM, 60% at concentrations <50µM, and ∼10% at concentrations >100 µM). The cytotoxicity assay occasionally can be extrapolated as positive at concentrations slightly higher than the maximum tested in the assay (*e.g.,* see Figure 1C). In such cases, if the agonist/antagonist assay was positive within the assay’s concentration range and if higher concentrations were tested, then the observed activity would likely persist. However, as there are increasing signs of cytotoxicity, we nevertheless considered these cases as not active up to the cytotoxicity limit, as discussed with the curation rules that we established and applied to all assay results.

- Class labels of fitted models of activity were set to *Negative* if the activity class was assigned for the highest tested concentration and if in parallel at this concentration there is a cytotoxicity signal above the background noise (but below the cytotoxicity limit). This rule was used as a shorthand for extrapolation of the cytotoxicity signal to cytotoxicity limit and its fully defined comparison with the activity signal using our special points ratio procedure (see Supplementary Figure 6).

#### 2.1.3 Data augmentation and dataset mergers

To be consistent with the curated subset of Tox21 *in vitro* data, data extracted from Binding DB was filtered down to records expressed as EC50 and IC50, because these data were obtained from cell-based *in vitro* assays, thus compatible with the Tox21 curated subset. The original data sources were not further investigated for veracity, consistency or data quality, because of the high number of data and because the peer-review of the BindingDB and primary sources were considered trustful. We included records described by EC50 or IC50 lower than 100 nM in our modelling dataset. According to descriptors collected from the curated Tox21 subset, we estimated a risk of 2% that a compound is cytotoxic when it is active *in vitro* at <100 nM. The threshold value was deduced from the Tox21 GH3 cell-based cytotoxicity assays where it was identified that cytotoxicity frequently started to occur at or above concentration of ca. 1 μM (see Supplementary Table S7, ∼10% of cytotoxicity labels given for concentrations <3 µM). The 100 nM threshold was obtained by further applying a safety factor of 10.

Finally, after data augmentation of the curated Tox21 dataset with the BindingDB database values, all compounds (with the same InChI) tested more than once and presenting divergent activity class labels (active and inactive) were filtered out from the curated dataset. Finally, after removal of salt counter-ions from all entries in the dataset, the following additional filters were applied: keep compounds represented by a single major constituent (mono-constituent like), and compounds containing atoms outside of the following list: Li, Be, C, N, O, F, Na, Mg, P, S, Cl, K, Ca, Se, Br, I.

As the BindingDB data was not systematically labelled as either agonist or antagonist, Tox21 data in the form of “agonist”, “antagonist” and “inactive” were relabelled as only two classes: “active” (grouping “agonists” and “antagonists”) and “inactive”. Several limitations also precluded the separation of the molecules into dedicate datasets for the agonist and the antagonist mechanisms of action. First, the Tox21 derived data inherently merges TRα and TRβ signals. Without the ability to distinguish between these biological targets, it is impossible to exclude mixed modes of action, wherein a molecule might exhibit activating influence on one receptor subtype and inhibiting influence on the other. Furthermore, partial or weak activators often present as competitive inhibitors when monitored in antagonist *in vitro* assays (such as TOX21_TR_Antagonist), making strict assignment to specific mechanistic training sets highly uncertain.

The final dataset is composed of 6,340 substances, of which 6,049 inactive substances and 291 active substances (containing 12 positives in agonist assay and 36 positives in antagonist assay from the Tox21 TR dataset, and 243 additional TR active substances from BindingDB). The dataset was highly imbalanced, with 95.4% of the data representing the inactive class and only 4.6% representing the active class. Given the small number of identified agonists and antagonists, it proved not feasible to train a model with these as separate classes, thus also supporting the choice of merging these two classes as “active”, and the choice of augmenting the dataset with BindingDB data despite the subsequent uncertainty in data quality.

### 2.2 Data-splitting procedure

All dataset partitions were made with in-house Python algorithms employing RDKit (Landrum et al., 2023) functionalities including the popular algorithms Butina clustering (Butina, 1999) and Kennard-Stone sampling (Kennard and Stone, 1969). Pairwise molecular similarities were computed using KREATiS′ proprietary hybrid 1D/2D fingerprint that is a modified version of PubChem fingerprint inspired by the implementation in PyBioMed (Dong et al., 2018) $T_{c}$ Similarity was quantified by the continuous Tanimoto coefficient ( $T_{c}$ ) (Tanimoto, 1958).

#### 2.2.1 Split Level 1 - Hierarchical butina clustering

**FIGURE 2:**
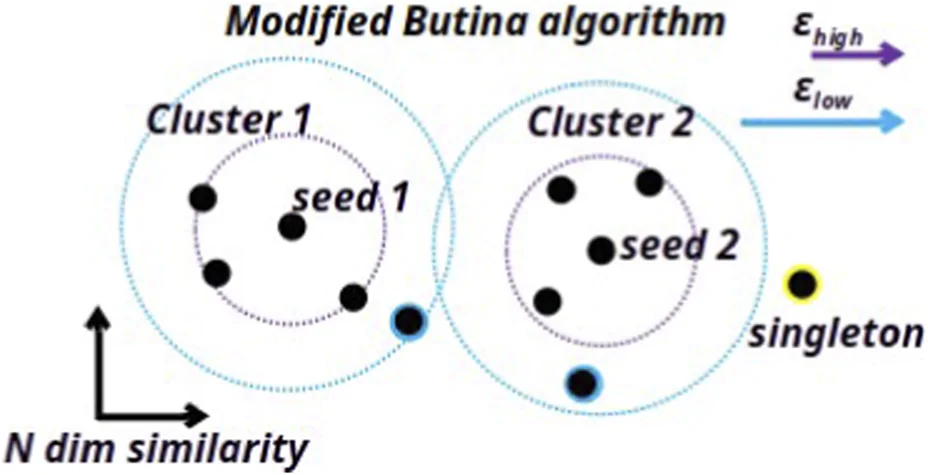
Iterative scheme employing Butina algorithm.

This methodology is schematised in Figure 2.

1. Given the pairwise similarity $T_{c}$ , we assume working in the metric space $\left(X,T_{c}\right)$ where, a pairwise distance is defined as:
$$d_{T_{ij}}\leftarrow 1-T_{c}\left(x_{i},x_{j}\right)\;\forall i\neq j$$
  Entries are merged in the same cluster when their pairwise distance is below an arbitrary defined threshold ε . The threshold at the first iteration of the Butina clustering was set to
$$\varepsilon_{high}=1-0.99,$$
  and the maximum distance at which Butina clustering would be performed was set to
$$\varepsilon_{low}=1-0.65.$$
2. At initialisation step t=0 , all entries are part of their own cluster, *e.g.*, all entries are singletons:
$$S_{t}\leftarrow {\left\{singleton_{i},\ldots ,singleton_{n}\right\}}_{t}\in X$$
3. The first Butina clustering step ( t ):
$$\varepsilon \leftarrow \varepsilon_{high}$$
$$D_{t}\leftarrow Butina\left(S_{t},\varepsilon \right)$$
  We distinguish the clusters by their cardinality. Multimember clusters
$$C_{t}\leftarrow {\left\{cluster_{1},\ldots ,cluster_{n}\right\}}_{t}\in D_{t}\;|\;card\left(cluster_{i}\right)>1,$$
  and singleton clusters
$$W_{t}\leftarrow {\left\{singleton_{1},\ldots ,singleton_{m}\right\}}_{t}\in D_{t}\;|\;card\left(cluster_{i}\right)\equiv 1$$
4. If there were no singleton clusters in $D_{t}$ then go to Step 9, or else update the iteration counter
t←t+1,
5. In order to accomplish efficient iterative clustering, we needed to verify the starting point of each subsequent Butina clustering run. We started by selecting central entries ( seeds ) from each cluster
$$B_{t}\leftarrow {\left\{seed_{1},\ldots ,seed_{n}\right\}}_{t}\in C_{t},$$
  and kept track of the remaining entries within each multimember cluster
$$D_{mask_{t}}\leftarrow C_{t}\backslash B_{t}$$
  Naturally, all singletons are seeds in their own clusters. In addition, purely for algorithmic purposes, we divided any cluster into singletons if it was composed of pairwise equidistant sets because it is not pertinent to select a cluster *seed* in such conditions. We consider the family $\left\{U_{\alpha }\right\}\in D_{t}$ of finite equilateral subsets, where each $U_{\alpha }$ satisfied $d_{T}\left(x_{i},x_{j}\right)=\delta_{\alpha }$ with ∀i≠j . Hence, we defined the merged set:
$$U_{t}≔\underset{\alpha \in A}{⋃}U_{\alpha }$$
  Ultimately, the total set of *seeds* for the next Butina clustering iteration became:
$$S_{t}\leftarrow B_{t}\cup U_{t}\cup W_{t}$$
6. We performed the next Butina clustering iteration:
ε← ε+0.01
$$D_{t}\leftarrow Butina\left(S_{t},\varepsilon \right)$$
7. We propagated the new cluster identifiers to the previously masked entries ( $D_{mask_{t}}$ ) through their association with $seed_{i}\in B_{t}$ .
8. If $\varepsilon <\varepsilon_{low}$ then we went to Step 4.
9. Result: all entries were either assigned to a cluster of similar molecules or remained a singleton (not similar to any other molecule up to cutoff $\varepsilon_{low}$ )

Contrarily to the classic Butina algorithm applied with a single $\varepsilon_{low}$ , the iterative scheme splits this toy-dataset into two clusters and a singleton. While, in the classic method there would be one large cluster whose seed would be the element highlighted in blue in Cluster 1, and includes the four closest elements, two from the depicted Cluster one and two from Cluster 2. Then, a singleton and two smaller clusters would be composed of two and three elements.

#### 2.2.2 Split Level 2 - Stratification by experimental activity

According to reviewed experimental THRBα/β activity, each cluster $\left\{C_{i}\right\}\in D_{t}$ (where *i* enumerate cluster identifiers) was split by positive activity (including both agonists and antagonists) class label P , and negative activity class label N :

$$P_{i}\leftarrow C_{i}\backslash N_{i}$$

#### 2.2.3 Split Level 3 - Isolation of the external validation set

The rules listed in Table 1 were applied to subsets $P_{i}$ and $N_{i}$ independently:

**TABLE 1:** Data splitting strategy for external validation set depending on the size of clusters.

Cluster size	Rule applied	Destination pool
n = 1	1: Merge singleton clusters $B_{P}≔\underset{i}{⋃}P_{i}\left|\;\right.card\left(P_{i}\right)\equiv 1$ $B_{N}≔\underset{i}{⋃}N_{i}\left|\;\right.card\left(N_{i}\right)\equiv 1$ 2: Sort the matrix of pairwise $T_{c}$ describing each set B , to maximise $T_{c}$ at the beginning of matrix indices 3: Determine the index step required to select 20% of the matrix for external validation	Training; validation external
n = 2	Random selection of the external validation	Training; external validation
n ≥ 3	Kennard-stone ranking → most peripheral 80%	Training; validation pool
	Remaining 20%	External validation

After the preprocessing in Splitting Level 1 and Splitting Level 2, the desired exact splitting of the curated dataset in 20%:80% subsets (classical *Train/Test* splitting scheme) was not straight forward. An exact 20%:80% ratio is only possible if all $P_{i}$ and $N_{i}$ clusters fulfil simultaneously the conditions:

$$card\left(P_{i}\right)\equiv 0\;\mathrm{mod}\;5$$

$$card\left(N_{i}\right)\equiv 0\;\mathrm{mod}\;5$$

Since these conditions were not exactly met, the global outcome of the *Train/Test* splitting workflow was.

- Prospective external validation set ≈20% (immediately locked)
- Training + validation pool ≈80%

#### 2.2.4 Split Level 4 - Creation of final training and internal validation sets

After isolation of the *Train* subset (Split Level 3), the procedure described in Split Level 3 was applied on the *Train* subset to isolate a validation pool, aiming at the splitting 80%:20%. Eventually the exact desired splitting ratio was not possible to achieve due to the fact that some $P_{i}$ and $N_{i}$ clusters have less than ten members.

Final global partition (typical values):

- Final training set 64% of original dataset
- Internal validation set 16% of original dataset
- Prospective external validation set 20% (fully independent, never used in Levels 1–4 operations on the other subsets)

### 2.3 Calculation of molecular fingerprints

Several molecular fingerprints were computed for each molecule using a combination of cheminformatics libraries. Substructure-based fingerprints (*e.g.,* PubChem, MACCS keys) were generated using PyBioMed, which internally relies on RDKit for molecular handling. Circular fingerprints such as ECFP6 and FCFP6 were computed directly with RDKit, using the Morgan algorithm and appropriate atom invariant definitions (Landrum et al., 2025). Additional fingerprints commonly used in QSAR modelling, such as complexity-related or topological descriptors, were also included depending on the model requirements.

A customised version of the PubChem fingerprint was developed. Starting from the implementation in PyBioMed, the features-counting scheme was adjusted. This fingerprint was used in the dataset splitting framework.

Each fingerprint type captures different structural or functional aspects of the molecule, providing complementary representations for machine learning.

### 2.4 Model building methods

#### 2.4.1 SVM model

Support Vector Machines (SVMs) are supervised learning techniques widely used for classification tasks. First, Introduced by Vapnik and colleagues (Cortes and Vapnik, 1995), SVMs aim to identify an optimal decision boundary, a hyperplane, which separates the data into distinct classes (Burges, 1998). However, real-world datasets are often not linearly separable in their original feature space. To address this, SVMs use a kernel, a mathematical function that measures similarity between points and implicitly maps the data into a higher-dimensional feature space in which a linear separation becomes possible. In the present model, the following hyperparameters were fine-tuned:

- C: This parameter controls how much the model allows for errors during training. By setting C to a lower value, the model focuses on creating a wider margin between the classes, even if it means misclassifying some points. This helps avoid overfitting and makes the model more general.
- Gamma: Gamma is particularly critical for nonlinear kernels, as it governs the flexibility and smoothness of the decision surface.
- Kernel: Specifies the type of transformation applied to the data to enable the model to capture linear or nonlinear relationships depending on the problem.

#### 2.4.2 Optimisation of model hyperparameters

The SVM hyperparameters, namely, the penalty parameter C and the kernel parameter γ, were optimised using grid search combined with five-fold cross-validation on the training set, where the five folds were generated using the splitting method provided by KREATiS, in order to identify the configuration offering the best predictive performance. Once the optimal hyperparameter configuration was identified, the model was evaluated on the internal validation set, and the best model was selected based on its performance on this validation set.

The optimisation was performed using the GridSearchCV function from Scikit-learn (Pedregosa et al., 2011), a widely used Python library for machine learning. This approach allows for the systematic exploration of a predefined parameter grid while evaluating each configuration via cross-validation, thus ensuring improved model performance while preserving its generalisation ability.

The optimisation process for a binary classification (active or inactive) also evaluated different types of molecular fingerprints, including substructure-based fingerprints (*e.g.*, PubChem and MACCS keys) and circular fingerprints (*e.g.*, Feature-Class Fingerprints FCFP6 and Extended-Connectivity Fingerprints ECFP6). The best predictive performance was obtained using circular fingerprints (FCFP6) as input features with C = 0.1 and kernel = “linear”. During the grid search, the F1-score was used as the evaluation metric to select the optimal hyperparameter configuration, ensuring a balanced assessment of both precision and recall.

### 2.5 Evaluation of model performance

To assess the robustness and unbiased predictive performance of the models, five-fold cross-validation and external validation were conducted. The predictive performance and robustness of the model were evaluated using a range of metrics, including classification accuracy (CA), sensitivity (SE), specificity (SP), precision, F1 score, and balanced accuracy (BA), as shown in Equations 1–6. In this study, an active compound misclassified as non-active is defined as a false negative (FN), whereas a non-active compound misclassified as active is defined as a false positive (FP). Correctly classified active and non-active compounds are referred to as true positives (TP) and true negatives (TN), respectively. Sensitivity and specificity correspond, respectively, to the proportion of correctly identified active compounds among all active compounds and the proportion of correctly identified non-active compounds among all non-active compounds. Classification accuracy represents the overall proportion of correctly classified compounds.

The formulas used to calculate these metrics are given below:

$$SE=TP/\left(TP+FN\right)$$ (1)

$$SP=TN/\left(TN+FP\right)$$ (2)

$$CA=\left(TP+TN\right)/\left(TP+TN+FP+FN\right)$$ (3)

$$Precision=TP/\left(TP+FP\right)$$ (4)

$$BA=\left(SE+SP\right)/2$$ (5)

$$F1=2*TP/\left(2*TP+FP+FN\right)$$ (6)

Additionally, F1 score and precision were calculated separately for the 1 (active) and 0 (inactive) to provide a more detailed evaluation of model performance in imbalanced datasets.

In addition, the receiver operating characteristic (ROC) curve and the precision-recall (PR) curve were used to assess the performance of the binary classification model. An area under the ROC curve (AUC-ROC) or PR curve (AUC-PR) of one indicates a perfect classifier, whereas a value of 0.5 for AUC-ROC (or a low AUC-PR) indicates poor discriminative ability.

### 2.6 The combined applicability domain and reliability assessment model

In this present work, “the combined applicability domain and reliability assessment model” refers to a combination of the applicability domain (AD) and the reliability assessment of the SVM model’s predictions.

#### 2.6.1 Assessment method for applicability domain

The applicability domain defines the region of chemical space and response space in which a model can make reliable predictions. Two complementary approaches were used to assess the domain:

1. Assessment of structural domain using Atom-Centred Fragmentation: For each molecule in the training set, atom-centred molecular fragments were generated and stored using RDKit. Each fragment included all atoms within a topological radius of 1, capturing the local chemical environment. To assess whether a new compound falls within the structural domain of the training set, its fragments are generated in the same way and compared against the stored training fragments. If all fragments are present in the training set, the compound is considered within the structural domain of the model.
2. Assessment of the descriptors domain using the k-nearest neighbours: To define the applicability domain based on molecular descriptors, the k-nearest neighbours (kNN) method first was calculated, for each compound of the training set, the average distance to its k nearest neighbours. From these average distances, a reference value was then determined using the interquartile range of the distances, as proposed by Sahigara et al. (2013):

$$\tilde{d}\left(k\right)=Q_{3}\left(\bar{d}\left(k\right)\right)+1.5\times \left[Q_{3}\left(\bar{d}\left(k\right)\right)-Q_{1}\left(\bar{d}\left(k\right)\right)\right]$$

where:

-
$\bar{d}\left(k\right)$ is the average distance to the k nearest neighbours.
- Q1 and Q3 are the 1st and 3rd quartiles, respectively, of the distances $\bar{d}\left(k\right)$ .
-
$\tilde{d}\left(k\right)$ is a reference value.

Next, the ordered distances of each i-th compound of the training set from all other n-1 compound of the training set were compared with the reference value. If the distance of the i-th sample from its j-th training neighbour (where1 ≤ j ≤ n-1 was less than or equal to the reference value, it was retained otherwise, it was discarded. Finally, each i-th compound of the training set was associated with a threshold t_i_ which defined the width of its neighbourhood as:

$$K_{i}:\left\{D_{ij}\leq \sim d\left(k\right)\right\},\forall j=1,\ldots ,n\text{‐}1t_{i}=\frac{1}{K_{i}}\sum_{j=1}^{K_{i}}D_{ij}$$

For a test compound, its distance to all compounds of the training set was calculated and compared with the thresholds t_i_. If the distance to at least one compound of the training set was less than or equal to its local thresholds, the compound was considered inside the domain otherwise it is outside the domain.

More formally, given the training set (TS), for each test compound j, the decision rule was:

$$n_{j}=\sum_{i\in TS}^{b}1_{D_{ij}\leq t_{i}},j\in AD\Leftrightarrow n_{j}\geq 1$$

- When n_j_ = 0 (number of training set neighbours that are sufficiently close according to the threshold t_i_) the compound was considered outside the descriptor domain.

In conclusion, a compound was considered outside the applicability domain if it is identified as out-of-domain by either the descriptor-based domain or the atom-centred molecular fragment approach.

#### 2.6.2 Assessment method for the reliability of prediction

**FIGURE 3:**
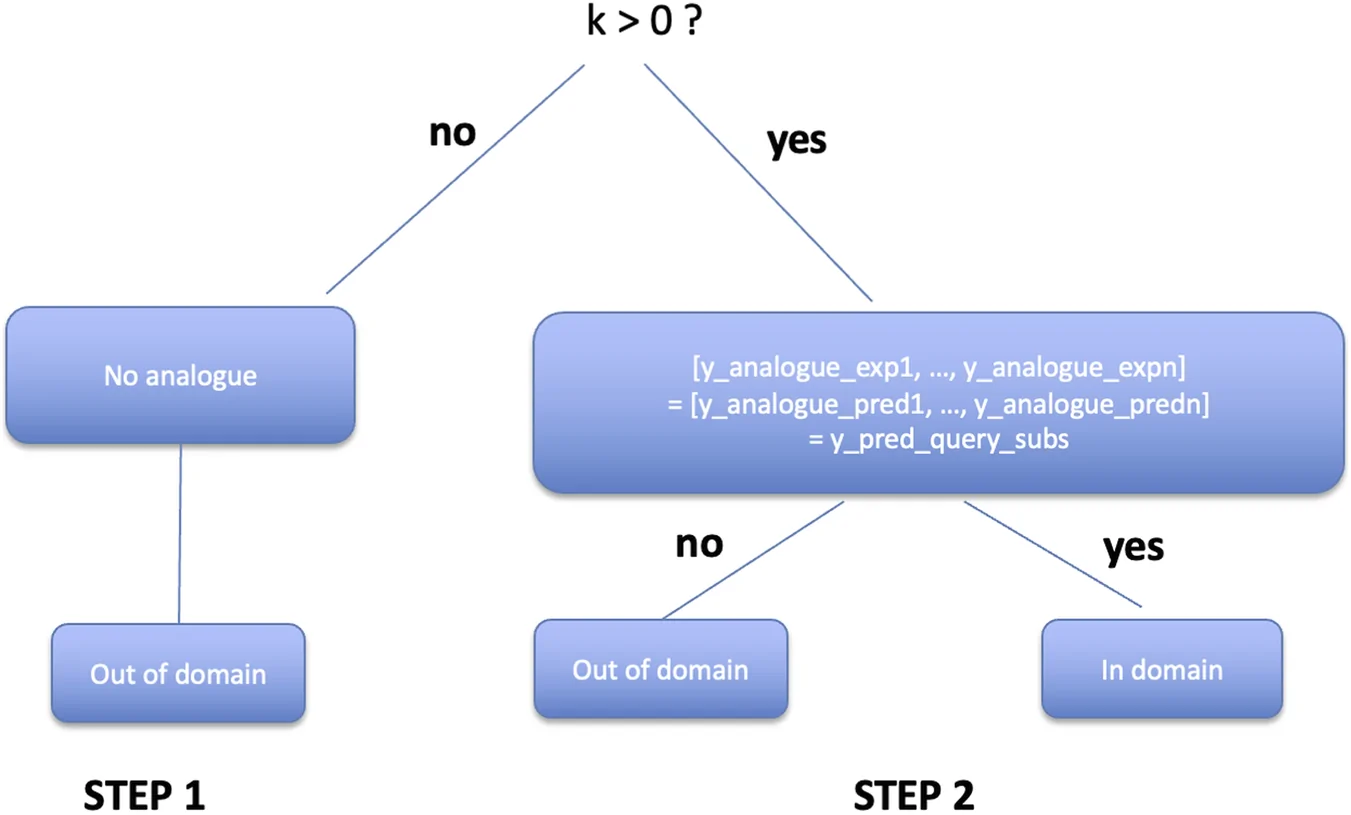
Assessment of prediction reliability.

The process was based on a binary classification model that categorises compounds prediction into two classes: “Reliable” and “Unreliable”. Analogues of the test compound were identified using a “k-Nearest Neighbours” approach based on the Tanimoto similarity index using a molecular fingerprint (Figure 3). Only compounds exceeding a predefined similarity threshold were retained. If no analogue was found, that is, if k = 0, the compound prediction was immediately classified as “Unreliable” (STEP 1).

When analogues were available, i.e., when k ≥ 1, two criteria were evaluated. The first criterion, accuracy, measured the agreement between the predicted and experimental values of the analogues. Accuracy was equal to one only if the predicted value of each analogue exactly matched its experimental value; otherwise, accuracy ≠ 1. The second criterion, concordance, reflected the consistency between the predicted value of the test compound and the experimental values of its analogues. Concordance was equal to one only if the predicted value of the test compound fully agreed with all experimental values of its analogues; otherwise, concordance ≠ 1. A compound prediction is classified as “Reliable only if both accuracy and concordance are equal to one; otherwise, it was classified as “unreliable” (STEP 2).

#### 2.6.3 Statistical evaluation of the combined applicability domain and reliability assessment model

To quantify the performance of this model, the exclusion rate and a confusion matrix were analysed. The ideal goal is to maintain a low exclusion rate while excluding all misclassified examples, while at the same time retaining correctly predicted compounds. This corresponds to maximising True Unreliable (TU), minimising False Unreliable (FU), and reducing the number of compounds considered out-of-domain by the applicability domain.

- exclusion rate: retains the majority of compounds to maximise coverage and allows the SVM model to make predictions across a wide range of molecules.

$$Exclusion\;rate=\frac{nb\;of\;compound\;Out‐of‐domain+TU+FU}{Total\;compounds}\times 100$$

The optimisation of the exclusion rate was performed using an internal validation set, which depends on both the descriptor-domain assessment and the reliability prediction assessment.

- Method of descriptor domain, the number of neighbours used to calculate the mean distance ${\bar{d}}_{\left(k\right)}$ was defined based on the FCFP6 molecular descriptors and was set to k = 3. In addition, K_i_≤3 was set as the value for calculating the local threshold.
- For the assessment method of reliability prediction, several molecular fingerprint types were evaluated, among which MACCS fingerprints showed the best performance. In this study, between one and three structural analogues (k = 1–3) with a Tanimoto similarity greater than 0.7 were considered to ensure reliable predictions.

The performance of the combined applicability domain and reliability assessment model was validated on a test set using the confusion matrix metrics (exclusion rate and proportion of mis-predicted compounds correctly flagged) (Table 2).

**TABLE 2:** Confusion matrix of the combined applicability domain and reliability assessment model.

Model outcome	In AD and predicted unreliable	In AD and predicted reliable
Mispredicted	True unreliable (TU) ✔ correctly flags a mispredicted molecule	False reliable (FR) × considers the molecule safe even though it is mispredicted
Correctly predicted	False unreliable (FU) × wrongly rejects a correctly predicted molecule	True reliable (TRe) ✔ correctly considers the molecule reliable

FR (False Reliable) represents the most critical cases where molecules are mis-predicted by the SVM but not detected so by the workflow, posing a potential risk for model reliability. FU (False Unreliable) are molecules correctly predicted by SVM but rejected by the workflow, which reduces model coverage. TU (True Unreliable) and TRe (True Reliable) correspond to correct decisions, indicating reliable detection.

## 3 Results

### 3.1 Curation method

**FIGURE 4:**
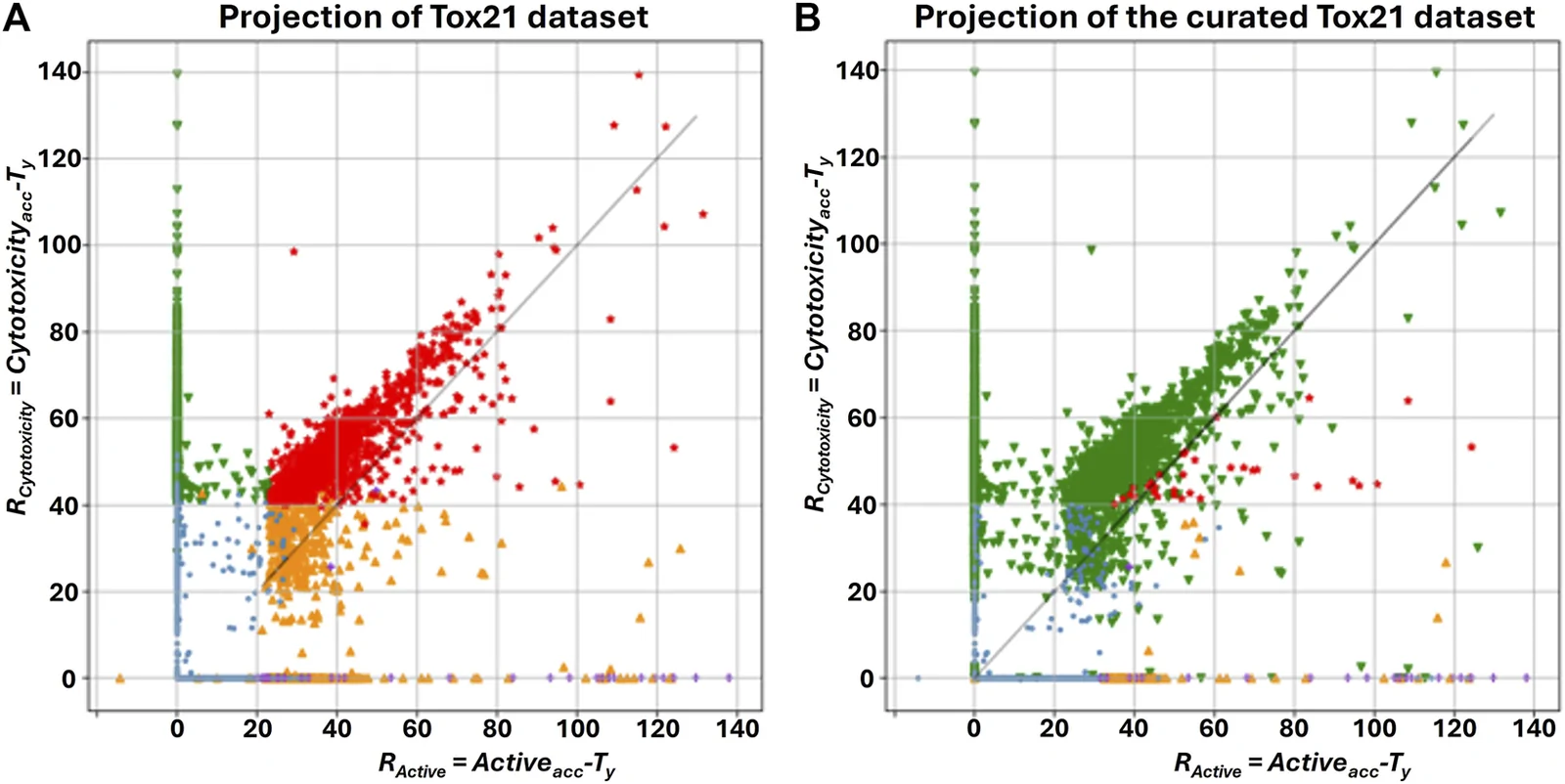
Overview of the input and the curated dataset after the automated curation. Entries coordinates are plotted as residuals (R) of activity or cytotoxicity in respect to a linear model of ascending activity (T). The colouring scheme aligns with the class labelling for activity allowed to tested samples in the Tox21 dataset: Agonist (purple), Antagonist (orange), Cytotoxic (green), Active (agonist or antagonist) and Cytotoxic (red), Not Active and Not Cytotoxic (blue). **(A)** Input dataset after transformation into R Cartesian coordinates. **(B)** The dataset after curation of the class labels the exact coordinates in R space computed from the initial data were preserved.

The standardisation of input structures discarded 434 entries (associated with activity measures) due to inability to process a chemical structure into an *in silico* object. Moreover; 1816 entries were filtered out because remaining multi-constituents after salt removal and/or contained atoms not permitted by the defined list (2.1.3). The dataset obtained after the automatic curation workflow had significantly lower number of entries labelled *Agonist* and/or *Antagonist*, compared to the original dataset (Table 3). The projection of the dataset before and after curation clearly demonstrates the distribution of active and cytotoxic labelled samples in both variants of the dataset (Figure 4). This massive shift from the “active” class to the “inactive” class stems from the fact that the original class labels in the Tox21 database are purely based on individual curve-fitting of agonist and antagonist assays, without comparison with corresponding cytotoxicity curves.

**TABLE 3:** Results of the automated curation method.

Assay	Original number of substances	After automated comparison with cytotoxicity curves	Reduced by unique and defined molecules without conflicting samples	After merging with BindingDB data and removal of multi-constituents and forbidden atoms
TOX21_TR_LUC _GH3_Agonist	35 active 7,947 inactive Total: 7,982	25 active 7,954 inactive Total: 7,979	21 active 7,924 inactive Total: 7,945	291 active 6,049 inactive Total: 6,340
TOX21_TR_LUC _GH3_Antagonist	2,318 active 5,846 inactive Total: 8,164	140 active 7,872 inactive Total: 8,012	103 active 7,809 inactive Total: 7,912	

After data augmentation with positive entries from BindingDB, the final number of substances in each class was as follows: 291 substances active on thyroid receptors *in vitro* and 6049 substances inactive on thyroid receptors *in vitro*.

### 3.2 Splitting method

The optimisation of the splitting procedure was made in respect to the classic Butina clustering method (detailed comparison not shown). For different clustering thresholds in the range 0.5≤ε≤0.35 , the classical method issued fewer singletons than the modified scheme; the classical method formed larger clusters, while the number of clusters issued by the iterative method appeared to converge. When examined by human experts, often the large clusters from the iterative method appeared much more chemically coherent in respect to equivalent clusters (both methods grouped substances around the same seeds) resulting from the classical algorithm, where substances seen as chemically not compatible with the rest of the cluster were present.

The distribution of the reference dataset among the *Train* and *External* validation subsets is detailed in Table 4. The counts demonstrated the severe disbalance between active and inactive class, 5.84% vs. 94.16% within the 6,351 entries available.

**TABLE 4:** Counts by subset during the dataset splitting process.

Split stage	Activity class label	Nb. entries	Nb. clusters	Nb. Singletons	%train set*	% internal validation* set	% external validation set*
Level 1	Active and inactive	6351	2351	593	N/A	N/A	N/A
Level 2	Active	371	173	88	N/A	N/A	N/A
Level 2	Inactive	5980	2178	505	N/A	N/A	N/A
Level 3	Active	371	173	88	74.93	N/A	25.07
Level 3	Inactive	5980	2178	505	71.20	N/A	28.80
Level 4	Active	371	173	88	56.06	18.87	25.07
Level 4	Inactive	5980	2178	505	52.54	18.66	28.80
Level 4	Active	371	173	88	3.28^	1.10^	1.46^
Level 4	Inactive	5980	2178	505	49.47^	17.57^	27.1^

(*) as fraction of entries in subsets at Level 2, (^) as fraction of entries of the total set at Level 1.

### 3.3 SVM hyperparameter optimisation for optimal model performance

A set of different molecular fingerprints, including MACCS keys, PubChem, FCFP6, and CFFP6, was employed in the present SVM model to classify substances as active (class 0) or inactive (class 1). The results obtained from the internal validation set are summarised in Supplementary Table 1. In this study, all fingerprints demonstrated good performance. However, the FCFP6 fingerprint, which achieved superior results, particularly in terms of sensitivity, was chosen for the optimal model. FCFP6 fingerprints are also considered advantageous, as they provide additional information about molecular functions that ECFP6 fingerprints may not capture (Supplementary Table 1).

### 3.4 Dataset representativity and consistency analysis

**FIGURE 5:**
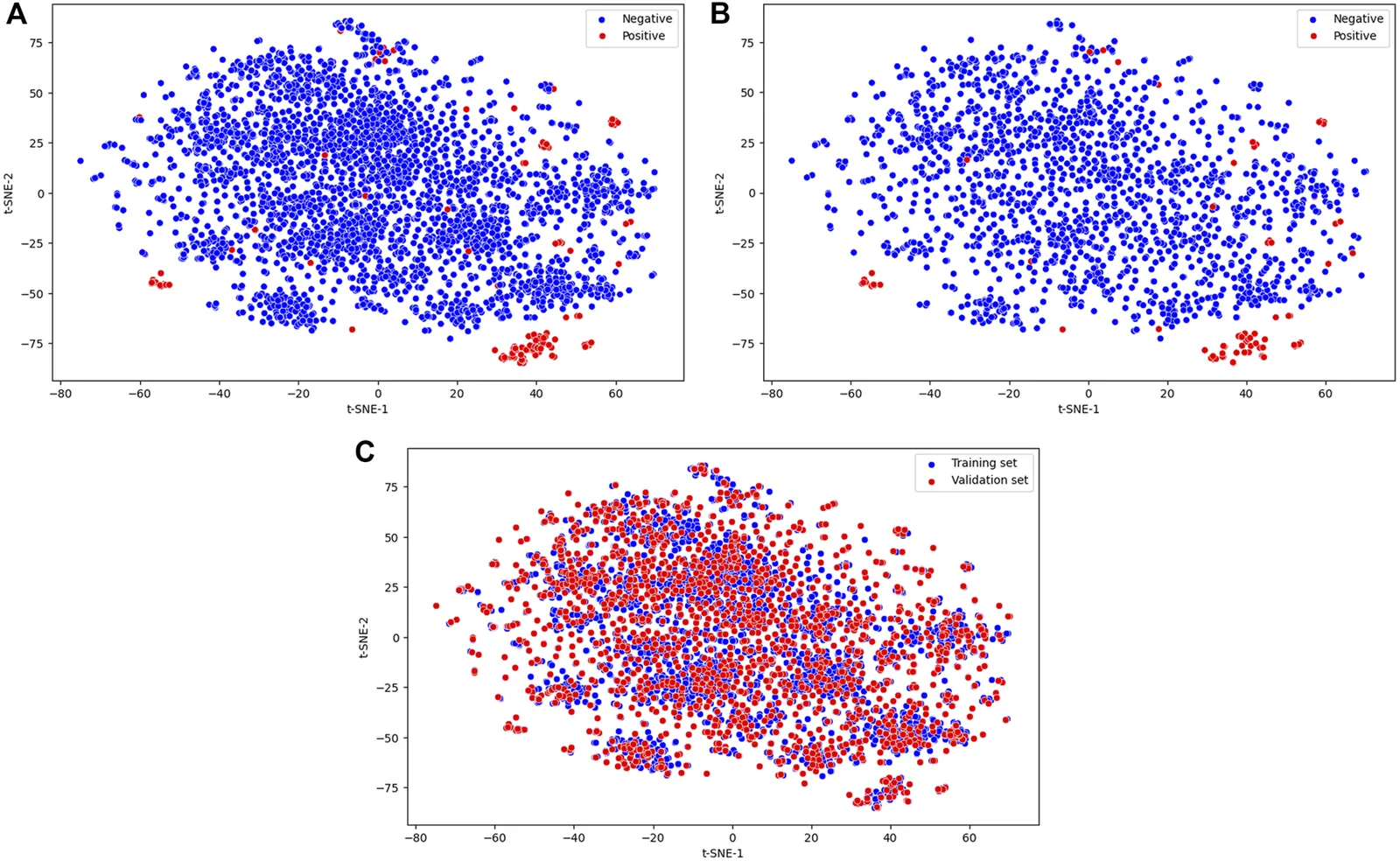
t-SNE projection visualising the clustering of molecules in the training and external validation sets. **(A)** Training set. **(B)** External validation set. **(C)** Training set and External validation set.

A t-SNE projection was performed on the training set, the test set, and the combined training + test sets (Figure 5). The visualisation clearly showed that active compounds were well separated from inactive ones in the model’s descriptor space, which explained the model’s strong ability to discriminate between the two classes. Moreover, training set and internal validation set points consistently overlapped for both classes, confirming that the test set was structurally representative of the training data in terms of model descriptors and chemical space coverage.

### 3.5 Statistics of the training set

A confusion matrix was generated for the training set, comprising 3,344 substances, to evaluate the model’s performance (Supplementary Table 2). The corresponding set of performance metrics is summarised in Table 5. The model correctly predicted all examples in the majority class (3,193 substances) while effectively detecting the minority class, with only 12 false negatives out of 151 instances. This resulted in a sensitivity of 92.05%, a specificity of 100%, a precision (positive predictive value) of 100%, and a negative predictive value (NPV) of 99.63%. These results demonstrate a good balance between sensitivity and specificity with high reliability in predictions for both classes.

**TABLE 6:** Confusion matrix for the combined applicability domain and reliability assessment model.

Model outcome	In AD and predicted unreliable	In AD and predicted reliable	Total
Mispredicted	7	5	12
Correctly predicted	177	1512	1689
Total	184	1517	1701

In contrast, a naïve baseline model that always predicts the majority (inactive) class would achieve an accuracy of 95.48% (3,193/3,344), which simply reflects the class proportion. The proposed model, with an accuracy of 99.64%, substantially outperforms this baseline and more importantly successfully identifies 92.05% of the rare active compounds while producing zero false positives. This clearly demonstrates the model’s ability to effectively address the severe class imbalance.

The balanced accuracy of 96.03% further highlights the model’s robust performance under class imbalance, as it assigns equal weight to both classes unlike standard accuracy.

The AUC-ROC score (99.91%) (Supplementary Figure 1) highlights the model’s ability to distinguish between classes across all decision thresholds, with a high value indicating excellent discriminative power. Meanwhile, the AUC-PR score (99.21%) (Supplementary Figure 2), which is particularly informative in imbalanced datasets, shows the precision-recall trade-off, confirming that the model excels in identifying the minority class without compromising precision.

### 3.6 Statistics of the external test set

The model was evaluated on 1,812 previously unseen compounds. It correctly classified 1,729 inactive compounds (2 false positives only) and 71 of 81 active compounds (sensitivity 87.65%), with 10 active compounds classified as false negatives (Supplementary Table 3). The high F1 scores for both classes demonstrate a good precision-recall balance, providing reliable identification of both active and inactive compounds while keeping the false positive rate low (Table 2).

### 3.7 Comprehensive summary of predictive performance and model generalisation

**FIGURE 6:**
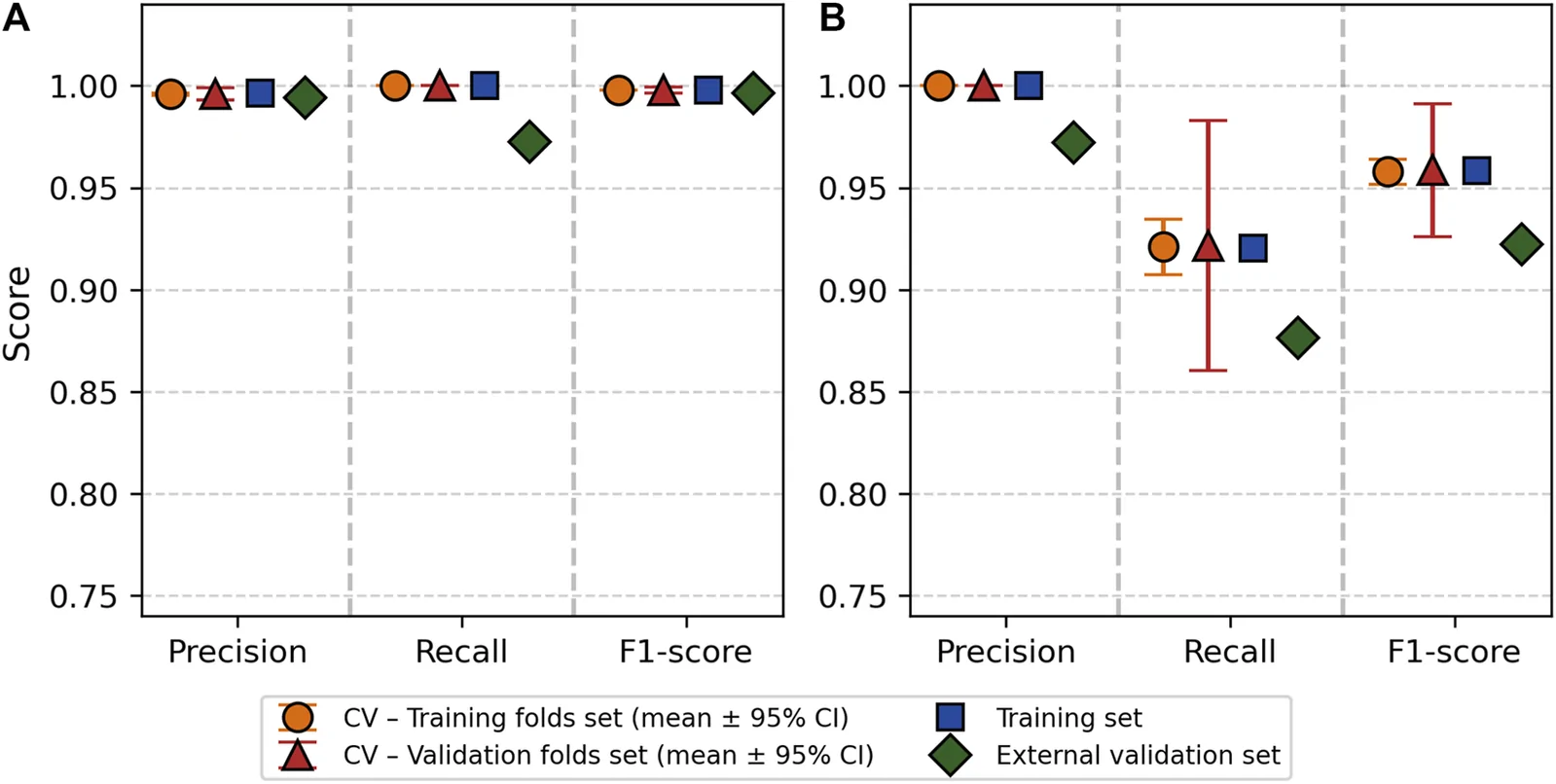
Classification performance metrics across all along the modelling workflow. **(A)** Metrics for Class 0 (Inactive). **(B)** Metrics for Class 1 (Active).

The statistics from the training folds set, validation folds set, the full training set, and the external validation set are presented in Figure 6. Of the training folds, all metrics (sensitivity, specificity, precision, and negative predictive value) exhibited very high mean values (∼92–100%) with narrow 95% confidence intervals (CI). Mean performance on the validation folds remained high and comparable to that observed on the training folds, indicating no substantial overfitting. The 95% confidence intervals for the metrics of the active class were wider, as expected in a highly imbalanced dataset combined with the limited number of folds, whereas those for the inactive class remained narrow. On the full training set, the same pattern was observed: excellent performance on the inactive class and slightly lower, yet still highly satisfactory, performance on the active class; all metrics fell within the confidence intervals derived from cross-validation. Finally, on the external validation set, performance metrics were very similar to those observed during cross-validation, with results for both the active and inactive classes falling entirely within the corresponding 95% confidence intervals.

### 3.8 Validation of the combined applicability domain and reliability assessment model on the external validation set

The applicability domain (AD) was computed on the external validation set containing 1,812 substances. When considering the descriptor domain alone, 111 substances were classified as outside of the descriptors domain, whereas all substances were considered in-domain according to the atom-centred fragment approach.

For the remaining substances, the method for assessing the reliability of predictions was applied. Of the 12 substances mispredicted by the SVM model, the prediction of only 7 substances was considered unreliable, while 5 remained reliable. Similarly, of the 1,649 substances correctly predicted by the SVM model, the prediction of 177 substances was classified as unreliable and 1,512 remained reliable (Table 6).

**TABLE 5:** Summary of model performance statistics for training set, cross-validation set with CIs and external validation before and after the combined applicability domain and reliability assessment model approach.

Statistics	Goodness-of-fit	Cross-validation (Training set mean [95% CI])	Cross-validation (Validation set mean [95% CI])	External validation (before AD)	External validation (after AD and reliability check)
Accuracy (%)	99.64	99.64 [99.50, 99.78]	99.62 [99.33, 99.91]	99.33	99.67
Balanced accuracy (%)	96.03	99.78 [99.72, 99.84]	99.81 [99.66, 99.96]	92.46	96.15
Sensitivity (%)	92.05	92.06 [90.61, 93.51]	92.17 [86.05, 98.29]	87.65	92.31
Precision (%)	100	100*	100*	97.26	100
F1-score - active (%)	95.86	95.80 [95.18, 96.42]	95.87 [92.60, 99.14]	92.21	96
Specificity (%)	100	100*	100*	99.42	100
Precision - inactive (class 0) (%)	99.63	99.62 [99.54, 99.69]	99.62 [99.38, 99.86]	99.42	99.66
F1-score - inactive (class 0) (%)	99.81	99.81 [99.77, 99.85]	99.81 [99.66, 99.96]	99.65	99.83
AUC-ROC (%)	99.91	N/A	N/A	96.09	N/A
AUC-PR (%)	99.21	N/A	N/A	92.38	N/A

Results are rounded to two decimal places. *Values of 100.00% indicate performances ≥ 99.95% (or equivalent after rounding).

The study shows that, among these 184 molecules for which predictions were either unreliable, or no confirmation could be obtained, 125 are singletons, meaning that they have no close analogues in the training set with a similarity threshold greater than 70%. They therefore do not share enough characteristics with the other molecules. Next, 59 of the 184 molecules were excluded during the second stage of assessment for the reliability of prediction.

The rate of molecules excluded by the model is 16.2%, while the exclusion rate for misclassified compounds is 58.33%.

The compounds related to a prediction identified as unreliable were removed from the test set prior to recalculating the statistics. The majority class (inactive) maintains an exceptional specificity of 100%, with 1452 true negatives without any false positive (Supplementary Table 4). The minority class (active) also shows a notable improvement with a sensitivity of 92.31% (59 true positives) and a reduction of false negatives to just 5. These results demonstrate that the model improved and is now better able to correctly identify the minority class while maintaining very high precision. Balanced accuracy, reflecting the balance between the two classes, also improves, rising from 92.46% to 96.15%, confirming an overall performance boost (Table 6).

## 4 Discussion

### 4.1 Curation and dataset splitting

The ultra-curation of the Tox21 TR dataset started with the standardisation of the description of chemicals. At this point of the workflow filters were applied to exclude metal-organic and multi-constituent entries in the dataset. Although numerous procedures are proposed in the literature, it appears that a “gold-standard” approach is not yet agreed upon, hence we chose to develop our model using the methods and data that are understood by the community. We are willing to address the limitations of these methodologies in the future, especially because many of the compounds discarded from the reference dataset are of interest to industry.

While our expert-driven curation related to cytotoxicity has the advantage of generating a dataset more representative of the “real” activity of chemicals to Thyroid Receptors, it results in difficulties comparing statistics between our model and other models for Thyroid Receptor-mediated activity. Most publicly available models are based on poorly curated datasets, often not fully accounting for the misleading cytotoxicity effect (*e.g.,* see Sapounidou et al., 2023). Thus, these models are trained to detect such cytotoxic substances as “active” and may achieve good statistics on a dataset with a similar level of curation. But such models would perform poorly on our curated dataset where many cytotoxic substances have been assigned as “inactive”, and conversely our model would obtain lower statistics on a less well curated dataset. The importance of creating a truly valid external dataset is thus highlighted.

Similarly, the choice that was taken to merge agonists and antagonists as a single “active” class (because of the low number of positives in each category and data augmentation with BindingDB) makes comparisons with other models more difficult, since other models often predict either agonist or antagonist. A kNN (k-Nearest Neighbours) method is envisaged as a further step to be applied to our model to distinguish between agonists and antagonists in the case where all nearest neighbours of a given test substance predicted as “active” would be identified as either agonists or antagonists.

On the other hand, the integration of Tox21 screening results with BindingDB records inherently introduces experimental heterogeneity, as the active molecules were evaluated across diverse assay types and laboratory conditions. While this variability presents a potential limitation regarding data noise, it also serves as a methodological strength: observing molecular activity across different experimental perspectives prevents the model from overfitting to the specific biological or technical biases of a single assay, thereby enhancing its generalizability. To mitigate the risks associated with this data merging, the dataset was rigorously curated to eliminate any compounds presenting conflicting activity records. Furthermore, it is important to note that the impact of this heterogeneity is confined strictly to the active minority class. The inactive class, which comprises 95.4% of the modelling dataset, was sourced exclusively from standardized Tox21 data and remains unaffected by cross-assay variance. Ultimately, we accepted the residual risk of potential experimental false positives within the active dataset, as this aligns with the conservative nature of our predictive modelling approach. Integrating the BindingDB data also lead to a wider applicability domain. Indeed, the Tox21 dataset is lacking pharmaceutically active substances that are on the other hand well represented in BindingDB.

Our novel iterative Butina clustering scheme proved to be a highly effective approach for partitioning structurally diverse chemical datasets. We did not include comparisons with random or kernel-based splitting strategies, as such methods do not enforce structural coherence and are therefore not appropriate baselines for evaluating clustering performance in highly heterogeneous chemical spaces. Hence, our objective was specifically to focus on improvements over the classical Butina algorithm within a chemically informed clustering framework. Instead, we benchmarked our approach against the classical Butina clustering method as implemented in RDKit.

The iterative scheme resulted in an early depletion of compounds surrounding singletons, highlighting a fundamental methodological difference between the two approaches. This behaviour accounts for the improved chemical coherence of the clusters produced by the iterative method, which, upon expert inspection, were substantially more consistent than those generated by the classical algorithm. In addition, the iterative approach yielded approximately 11% fewer singletons (678 substances) compared with the classical method (761 substances), thereby reducing the number of compounds that had to be assigned to training and test subsets in a quasi-random manner. Because singleton compounds are at high risk of being classified as outside the model’s applicability domain by the kNN-based approach, this reduction represents an additional and practically relevant advantage of the proposed scheme. While this behaviour warrants further investigation through application of the workflow to other datasets, the present results clearly indicate a methodological improvement over the classical Butina clustering strategy.

### 4.2 New version of Tox21 dataset

During the course of this work, the US EPA released invitroDB v4.1 (Feshuk et al., 2023, Center for Computational Toxicology US EPA ORD, 2023), which is based on the same raw data but with an updated data processing pipeline, compared to invitroDB v3.3 (EPA’s National Center for Computational Toxicology, 2020) that was used in the present work. Only a limited number of substances exhibited changes in activity class labels relative to the previous release (Supplementary Table S6), while the primary updates to the Tox21 TR dataset concerned the inclusion of additional fitted models and modifications to data representation. Despite these enhancements, we retained invitroDB v3.3, as our computational workflows were specifically designed for its data structures.

A key feature of our ultra-curationmethodology is the systematic reassignment of activity labels to Inactive when activity signals observed at concentrations comparable to those associated with significant cytotoxicity. Manual quality assessment of positive substances persisting after the automated curation, further incorporated external criteria including auto-fluorescence and well-documented assay biases. To our knowledge, this integrated and large-scale curation strategy has not been previously applied in the development of predictive models based on the Tox21 TR dataset and thus represents a substantial contribution to QSAR modelling of substances’ activity on the Thyroid Receptors.

### 4.3 Model and the combined applicability domain and reliability assessment model

Within the regulatory requirements framework, it is essential to have an unambiguous, transparent model supported by a clearly defined scope of applicability. Each step in the modelling process must therefore be explicitly documented, starting with the selection and description of molecular descriptors. Taking these requirements into account, the SVM model for classifying compounds, except organometallic and salt derivatives, as active or inactive not only demonstrated high predictive performance, but also excellent robustness when using FCFP6 fingerprints. In addition, the application of the combined applicability domain and reliability assessment model significantly improved the model’s performance, particularly for the identification of active compounds. Moreover, the overall exclusion rate of 16% is relatively low, demonstrating the model’s relevance and effectiveness, as it retains the majority of compounds while successfully identifying and filtering out unreliable predictions.

However, FCFP6 fingerprints may be more complex for regulatory evaluators to interpret, as they are based on an abstract representation of atomic motifs encoded as bits, with no direct correspondence to explicit chemical descriptors or easily identifiable functional groups. In this context, the use of predefined structural fingerprints (PubChem, MACCS, etc.) is often preferred to facilitate understanding by non-specialists and to enhance transparency during regulatory evaluation. The results presented in Supplementary Table 1 indicate that these fingerprints offer generally satisfactory performance but remain inferior to those obtained with FCFP6. However, they can be optimised, particularly through approaches based on genetic algorithms, to enhance their robustness and predictive efficiency.

In a screening context, where sensitivity and rapid identification of potentially active compounds are priorities, the model based on FCFP6 remains particularly relevant and effective.

The approach to defining the applicability domain (AD) is based on a combination of two complementary methods: one based on the structural domain, the other on the descriptors domain. The descriptor-based approach assesses whether a new compound is sufficiently represented in the local neighbourhood of the training set, thereby increasing confidence in the model’s predictions. Then, the reliability of predictions is assessed using three criteria: (i) the similarity of analogues, based on a predefined threshold and a fixed number of neighbours (k); (ii) accuracy, ensuring thatthe predicted value for each analogue exactly matches its experimental value; and (iii) concordance, by comparing the experimental values of the analogues with the predicted value for the query compound. This reliability assessment method is conservative, considering a prediction reliable only when all identified analogues strictly meet both the accuracy and concordance criteria (Figure 3). It should be noted that this methodology strictly follows the recommendations of the QSAR Applicability Framework (QAF) (OECD, 2024b), a regulatory framework designed to ensure that QSAR models provide reliable and relevant predictions.

Thus, the best performance for evaluating the combined applicability domain and reliability assessment model was obtained using MACCS molecular fingerprints, which are therefore recommended as the default option, particularly for non-expert users. However, for expert users, the methodology remains flexible: other, more specific types of fingerprints can be selected, and the number of neighbours (k) can be adjusted to recompute prediction reliability. Since MACCS key fingerprints provide a relatively general description of molecular similarity, the use of more detailed fingerprints and alternative k values may allow a more tailored and refined assessment of prediction reliability in specific applications. Although this strategy guarantees a high level of confidence, it leads to a very limited scope of applicability, restricting the number of compounds for which a prediction can be considered reliable. It would therefore be particularly relevant to introduce an overall reliability index, incorporating the three key parameters, similarity, accuracy, and concordance in a weighted form. Such an approach would allow the influence of each criterion to be adjusted based on the relevance of the analogues. A similar approach is implemented in the VEGA software to assess the reliability of predictions (Benfenati et al., 2013; Danieli et al., 2023).

## 5 Conclusion

In conclusion, despite the modelling challenges expected due to severe dataset disbalance, the ultra-curation and advanced dataset splitting provided high quality training opportunities for the SVM model to effectively combines predictive performance, robustness. Moreover, the rigorous definition of the domain of applicability completes the presented model with a conservative assessment of the reliability of predictions. Put together, the iVEMPS-THR complies with newest regulatory requirements for *in silico* predictions, being characterised with outstanding accuracy, and concordance it ensures a high level of confidence while offering methodological flexibility tailored to the needs of both expert and non-expert users.

## Data Availability

Publicly available datasets were analyzed in this study. This data can be found here: https://comptox.epa.gov/dashboard/; https://www.bindingdb.org/rwd/bind/index.jsp.

## References

[B1] Autrup H. Barile F. A. Berry S. C. Blaauboer B. J. Boobis A. Bolt H. (2020). Human exposure to synthetic endocrine disrupting chemicals (S-EDCs) is generally negligible as compared to natural compounds with higher or comparable endocrine activity: how to evaluate the risk of the S-EDCs? Arch. Toxicol. 94, 2549–2557. 10.1007/s00204-020-02800-8 32514609 PMC7367909

[B2] Axelstad M. Boberg J. Vinggaard A. M. Christiansen S. Hass U. (2013). Triclosan exposure reduces thyroxine levels in pregnant and lactating rat dams and in directly exposed offspring. Food Chem. Toxicol. 59, 534–540. 10.1016/j.fct.2013.06.048 23831729

[B3] Benfenati E. Manganaro A. Gini G. (2013). VEGA-QSAR: AI inside a platform for predictive toxicology. CEUR Workshop Proc. 1107. Available online at: https://ceur-ws.org/Vol-1107/paper8.pdf (Accessed July 14, 2026).

[B4] Brescia S. (2020). Thresholds of adversity and their applicability to endocrine disrupting chemicals. Crit. Rev. Toxicol. 50, 213–218. 10.1080/10408444.2020.1740973 32228218

[B5] Burges C. J. C. (1998). A tutorial on support vector machines for pattern recognition. Data Min. Knowl. Discov. 2 (2), 121–167. 10.1023/A:1009715923555

[B6] Butina D. (1999). Unsupervised data base clustering based on Daylight’s fingerprint and tanimoto similarity: a fast and automated way to cluster small and large data sets. J. Chem. Inf. Comput. Sci. 39, 747–750. 10.1021/ci9803381

[B81] Center for Computational Toxicology US EPA ORD (2023). ToxCast Database: invitrodb version 4.1. 10.23645/epacomptox.6062623.v11

[B7] Cortes C. Vapnik V. (1995). Support-vector networks. Mach. Learn. 20 (3), 273–297. 10.1007/BF00994018

[B8] Danieli A. Colombo E. Raitano G. Lombardo A. Roncaglioni A. Manganaro A. (2023). The VEGA tool to check the applicability domain gives greater confidence in the prediction of *in silico* models. Int. J. Mol. Sci. 24, 9894. 10.3390/ijms24129894 37373049 PMC10298077

[B9] Dong J. Yao Z. J. Zhang L. Luo F. Lin Q. Lu A. P. (2018). PyBioMed: a python library for various molecular representations of chemicals, proteins and DNAs and their interactions. J. Cheminformatics 10, 16. 10.1186/s13321-018-0270-2 29556758 PMC5861255

[B10] ECHA (European Chemicals Agency) (2024). Guidance on the Application of the CLP Criteria - Part 3: Health Hazards: Guidance to Regulation (EC) No 1272/2008 on Classification, Labelling and Packaging (CLP) of Substances and Mixtures. Version 5.0, November 2024. Helsinki: European Chemicals Agency. 10.2823/9350054

[B11] ECHA (European Chemicals Agency), EFSA (European Food Safety Authority) with the technical support of the Joint Research Centre (JRC), Andersson N. Arena M. Auteri D. Barmaz S. (2018). Guidance for the identification of endocrine disruptors in the context of regulations (EU) No 528/2012 and (EC) No 1107/2009. EFSA J. 16 (6), e05311. 10.2903/j.efsa.2018.5311 32625944 PMC7009395

[B80] EPA’s National Center for Computational Toxicology (2020). ToxCast Database (invitroDB). 10.23645/epacomptox.6062623.v5

[B12] Evangelista M. Papa E. (2025). A review of quantitative structure-activity relationship (QSAR) models to predict thyroid hormone system disruption by chemical substances. Toxics 13, 799. 10.3390/toxics13090799 41012420 PMC12474327

[B13] Feldt-Rasmussen U. Mathiesen E. R. (2011). Endocrine disorders in pregnancy: physiological and hormonal aspects of pregnancy. Best. Pract. Res. Clin. Endocrinol. Metab. 25, 875–884. 10.1016/j.beem.2011.07.004 22115163

[B14] Fernandez M. O. Bourguignon N. S. Arocena P. Rosa M. Libertun C. Lux-Lantos V. (2018). Neonatal exposure to bisphenol A alters the hypothalamic-pituitary-thyroid axis in female rats. Toxicol. Lett. 285, 81–86. 10.1016/j.toxlet.2017.12.029 29305326

[B46] Feshuk M. Kolaczkowski L. Dunham K. Davidson-Fritz S. E. Carstens K. E. Brown J. (2023). The ToxCast pipeline: updates to curve-fitting approaches and database structure. Front. Toxicol. 5, 1275980. 10.3389/ftox.2023.1275980 37808181 PMC10552852

[B15] Freitas J. Miller N. Mengeling B. J. Xia M. Huang R. Houck K. (2014). Identification of thyroid hormone receptor active compounds using a quantitative high-throughput screening platform. Curr. Chem. Genom. Transl. Med. 8, 36–46. 10.2174/2213988501408010036 24772387 PMC3999704

[B16] Kennard R. W. Stone L. A. (1969). Computer aided design of experiments. Technometrics 11, 137–148. 10.1080/00401706.1969.10490666

[B17] Klip H. Verloop J. van Gool J. D. Koster M. E. Burger C. W. van Leeuwen F. E. (2002). Hypospadias in sons of women exposed to diethylstilbestrol in utero: a cohort study. Lancet 359, 1102–1107. 10.1016/S0140-6736(02)08152-7 11943257

[B18] La Merrill M. A. Vandenberg L. N. Smith M. T. Goodson W. Browne P. Patisaul H. B. (2020). Consensus on the key characteristics of endocrine-disrupting chemicals as a basis for hazard identification. Nat. Rev. Endocrinol. 16, 45–57. 10.1038/s41574-019-0273-8 31719706 PMC6902641

[B19] Landrum G. Tosco P. Kelley B. Rodriguez R. Cosgrove D. Sriniker S. (2023). Rdkit/rdkit: 2023_09_2 (Q3 2023) release (Release_2023_09_2). Zenodo. Dataset/Software. 10.5281/zenodo.10099869

[B20] Landrum G. Tosco P. Kelley B. Rodriguez R. Cosgrove D. Vianello R. (2025). RDKit (version 2025_09_1). Zenodo. 10.5281/zenodo.591637

[B21] Liu T. Hwang L. Burley S. K. Nitsche C. I. Southan C. Walters W. P. (2025). BindingDB in 2024: a FAIR knowledgebase of protein-small molecule binding data. Nucleic Acids Res. 53, D1633–D1644. 10.1093/nar/gkae1075 39574417 PMC11701568

[B22] Marques A. C. Mariana M. Cairrao E. (2022). Triclosan and its consequences on the reproductive, cardiovascular and thyroid levels. Int. J. Mol. Sci. 23, 11427. 10.3390/ijms231911427 36232730 PMC9570035

[B23] Mustieles V. Balogh R. K. Axelstad M. Montazeri P. Márquez S. Vrijheid M. (2023). Benzophenone-3: comprehensive review of the toxicological and human evidence with meta-analysis of human biomonitoring studies. Environ. Int. 173, 107739. 10.1016/j.envint.2023.107739 36805158

[B24] National Center for Biotechnology Information (2014). PubChem bioassay record for AID 743064, source: Tox21. Available online at: https://pubchem.ncbi.nlm.nih.gov/bioassay/743064 (Accessed June 18, 2026).

[B25] Nc3Rs (2026). Available online at: https://nc3rs.org.uk/crackit/crack-it-challenges (Accessed January 26, 2026).

[B26] Norman A. W. Henry H. L. (2015). Hormones. 3rd Edition. Amsterdam: Elsevier Academic Press.

[B27] OECD (Organisation for Economic Co-operation and Development) (2024a). Thyroid *in vitro* Methods: Assessment Reports by the Thyroid Disruption Methods Expert Group - Reports Assessing the Validation Status of Assays from the EU-NETVAL Activities. OECD Series on Testing and Assessment. Paris: OECD Publishing. 10.1787/3786c75f-en

[B28] OECD (Organisation for Economic Co-operation and Development) (2024b). (Q)SAR Assessment Framework: Guidance for the Regulatory Assessment of (Quantitative) Structure Activity Relationship Models and Predictions, Second Edition. OECD Series on Testing and Assessment, No. 405. Paris: OECD Publishing. 10.1787/bbdac345-en

[B29] OECD (Organisation for Economic Co-operation and Development) (2025a). Report on the State of the Science to Address Endocrine Disrupters Under the Globally Harmonised System of Classification and Labelling. OECD Series on Testing and Assessment, No. 427. Paris: OECD Publishing. 10.1787/9aa7cd81-en

[B30] OECD (Organisation for Economic Co-operation and Development) (2025b). Test No. 422: Combined Repeated Dose Toxicity Study with the reproduction/developmental Toxicity Screening Test. OECD Guidelines for the Testing of Chemicals, Section 4: Health Effects. Paris: OECD Publishing. 10.1787/9789264264403-en

[B31] Paul-Friedman K. Martin M. Crofton K. M. Hsu C. W. Sakamuru S. Zhao J. (2019). Limited chemical structural diversity found to modulate thyroid hormone receptor in the Tox21 chemical library. Environ. Health Perspectives 127 (9), 97009. 10.1289/EHP5314 31566444 PMC6792352

[B32] Pedregosa F. Varoquaux G. Gramfort A. Michel V. Thirion B. Grisel O. (2011). Scikit-learn: machine learning in python. J. Mach. Learn. Res. 12, 2825–2830. Available online at: https://jmlr.org/papers/v12/pedregosa11a.html.

[B33] Robotti S. (2021). The heritable legacy of diethylstilbestrol: a bellwether for endocrine disruption in humans. Biol. Reprod. 105 (3), 687–689. 10.1093/biolre/ioab146 34402505

[B34] Sahigara F. Ballabio D. Todeschini R. Consonni V. (2013). Defining a novel k-nearest neighbours approach to assess the applicability domain of a QSAR model for reliable predictions. J. Cheminformatics 5, 27. 10.1186/1758-2946-5-27 23721648 PMC3679843

[B35] Sapounidou M. Norinder U. Andersson P. L. (2023). Predicting endocrine disruption using conformal prediction - a prioritization strategy to identify hazardous chemicals with confidence. Chem. Research Toxicology 36 (1), 53–65. 10.1021/acs.chemrestox.2c00267 36534483 PMC9846826

[B36] Sellami A. Réau M. Montes M. Lagarde N. (2022). Review of *in silico* studies dedicated to the nuclear receptor family: therapeutic prospects and toxicological concerns. Front. Endocrinol. 13, 986016. 10.3389/fendo.2022.986016 36176461 PMC9513233

[B37] Taha M. Marie A. M. Ahmed-Farid O. A. (2020). Combined approaches for evaluation of xenoestrogen neural toxicity and thyroid dysfunction: screening of oxido-nitrosative markers, DNA fragmentation, and biogenic amine degradation. J. Biochem. Mol. Toxicol. 34, e22521. 10.1002/jbt.22521 32492287

[B38] Tanimoto T. T. (1958). An Elementary Mathematical Theory of Classification and Prediction. New York: International Business Machines Corporation.

[B39] UNEP (United Nations Environment Programme) and WHO (World Health Organization) (2012). State of the Science of Endocrine Disrupting Chemicals 2012. Geneva: UNEP/WHO.

[B41] Vergauwen L. Bajard L. Tait S. Langezaal I. Sosnowska A. Roncaglioni A. (2024). A 2024 inventory of test methods relevant to thyroid hormone system disruption for human health and environmental regulatory hazard assessment. Open Res. Eur. 4, 242. 10.12688/openreseurope.18681.1 39931575 PMC11809485

[B42] WHO (World Health Organization) and IPCS (International Programme on Chemical Safety) (2002). Global assessment of the state-of-the-science of endocrine disruptors. Available online at: https://www.who.int/publications/i/item/WHO-PSC-EDC-02.2 (Accessed January 26, 2026).

[B43] Williams A. J. Grulke C. M. Edwards J. McEachran A. D. Mansouri K. Baker N. C. (2017). The CompTox chemistry dashboard: a community data resource for environmental chemistry. J. Cheminform. 9, 61. 10.1186/s13321-017-0247-6 29185060 PMC5705535

[B44] Yonchev D. Bajorath J. (2020). Inhibitor bias in luciferase-based luminescence assays. Future Sci. OA 6, FSO594. 10.2144/fsoa-2020-0081 32983562 PMC7491066

[B45] Zhang P. Yang M. Zeng L. Liu C. (2018). P38/trhr-Dependent regulation of TPO in thyroid cells contributes to the hypothyroidism of triclosan-treated rats. Cell Physiol. Biochem. 45 (4), 1303–1315. 10.1159/000487558 29462796

