# Supplementary material for: A new high-accuracy QSAR model based on ultra-curated data for predicting thyroid receptor-related endocrine activity
Source: Front Toxicol. 2026 Aug 20;8:1852502. doi: 10.3389/ftox.2026.1852502 (PMC13540040; doi:10.3389/ftox.2026.1852502)
Supplement: Supplementary file 1 [file DataSheet1.pdf]

Supplementary Material

1 Supplementary Figures and Tables

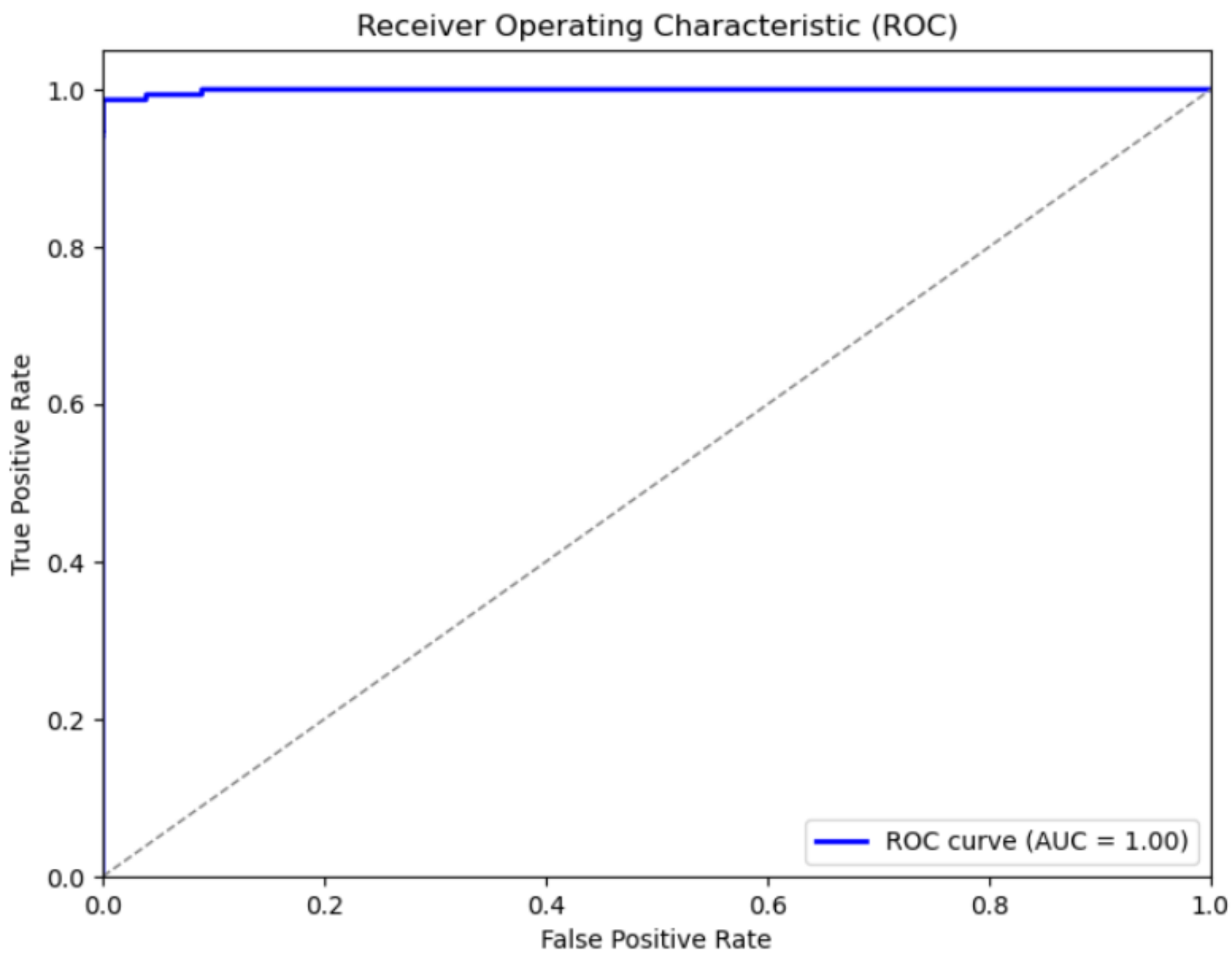

Supplementary Figure 1. Model performance on the training set assessed by the area under the receiver operating characteristic curve (AUC-ROC).

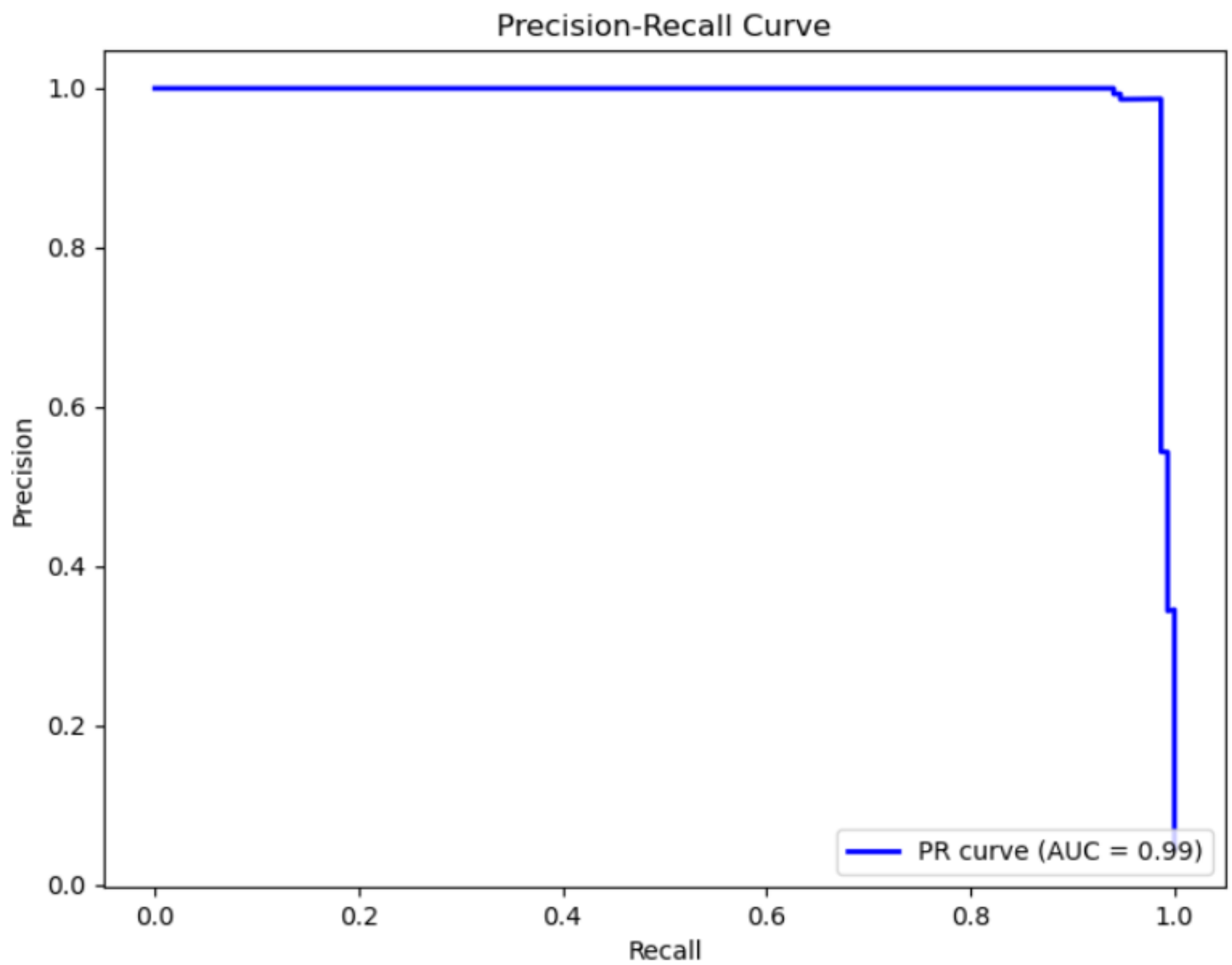

Supplementary Figure 2. Model performance on the training set assessed by the area under the precision–recall curve (AUC-PR).

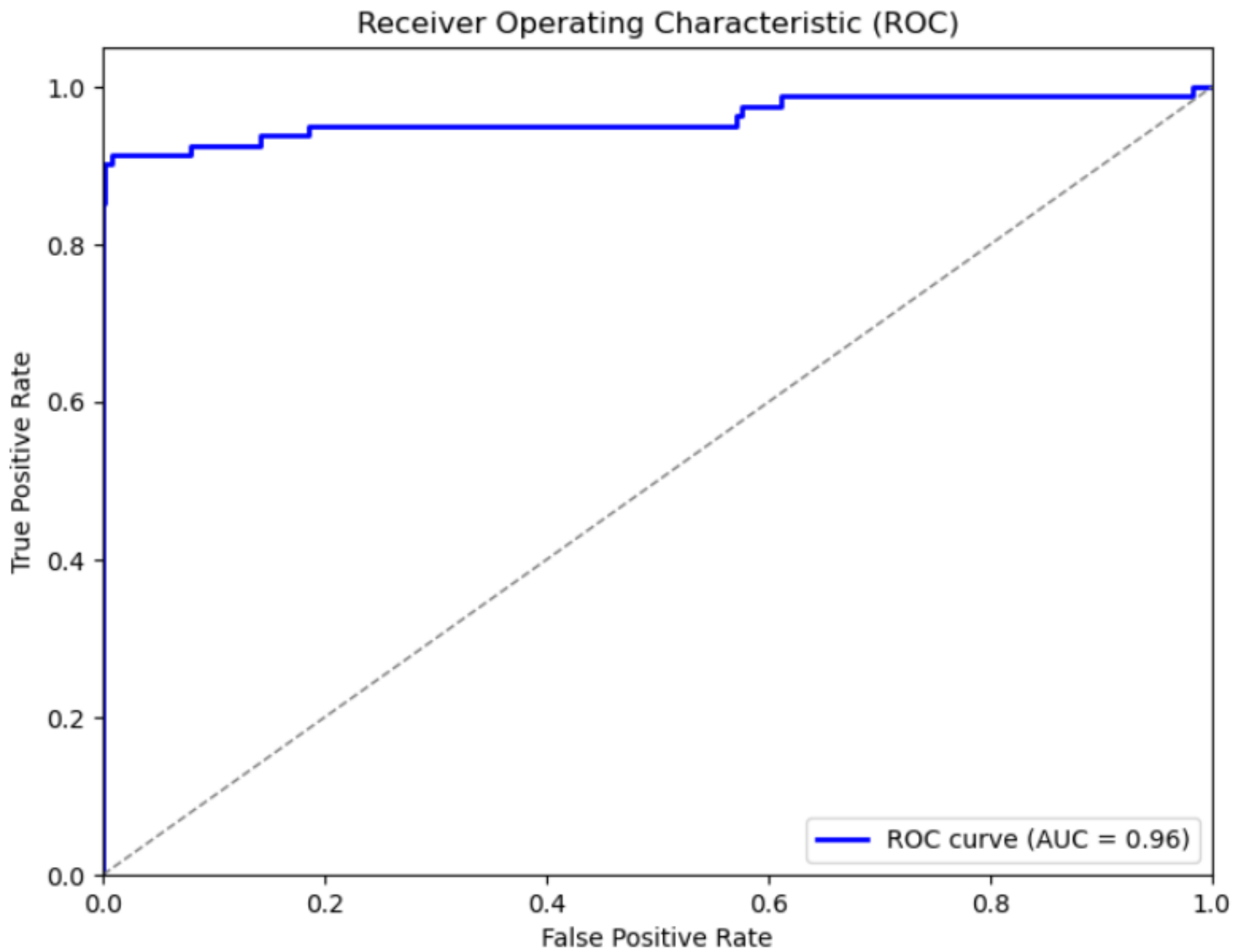

Supplementary Figure 3. Model performance on the test set assessed by the area under the receiver operating characteristic curve (AUC-ROC).

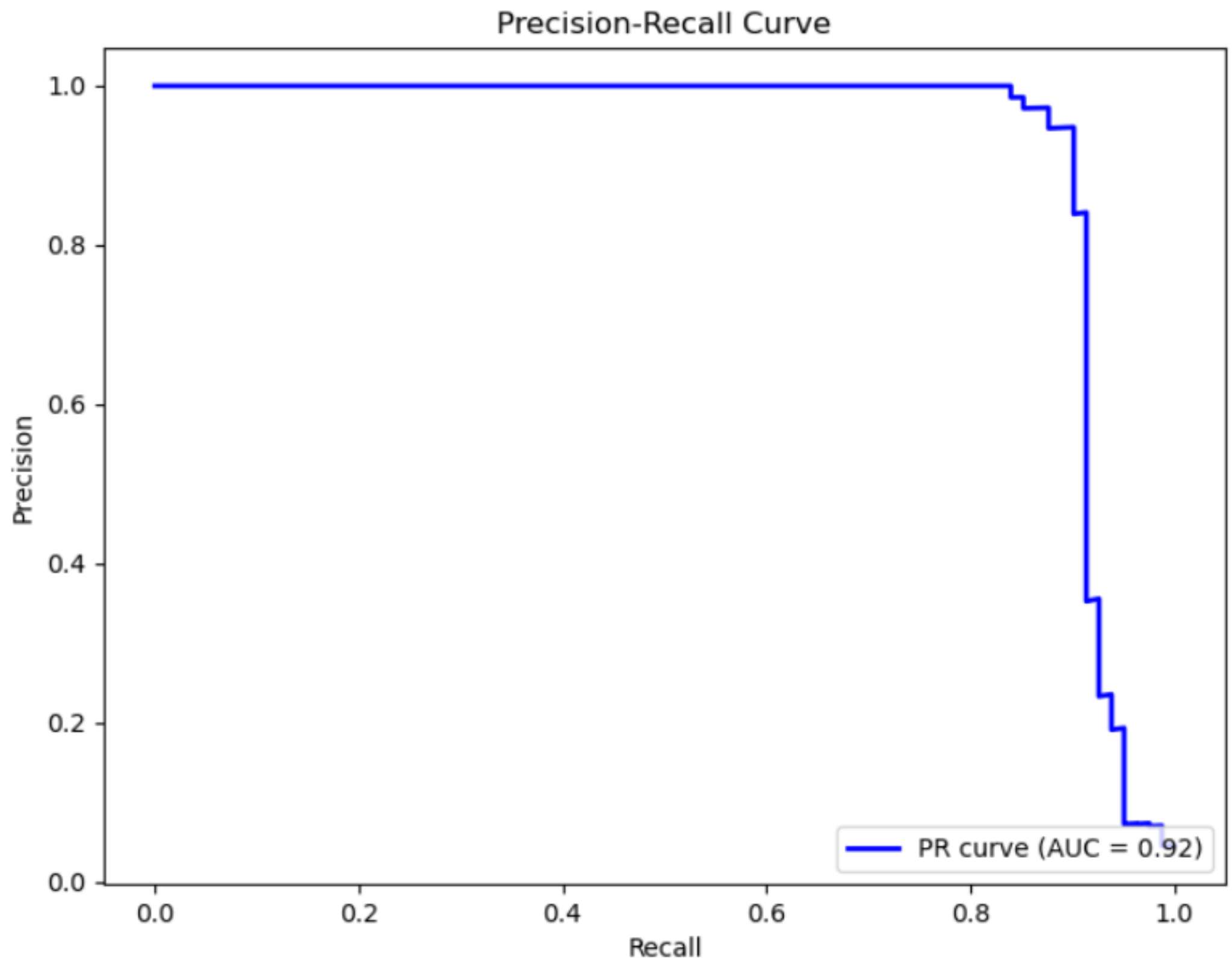

Supplementary Figure 4. Model performance on the training set assessed by the area under the precision–recall curve (AUC-PR).

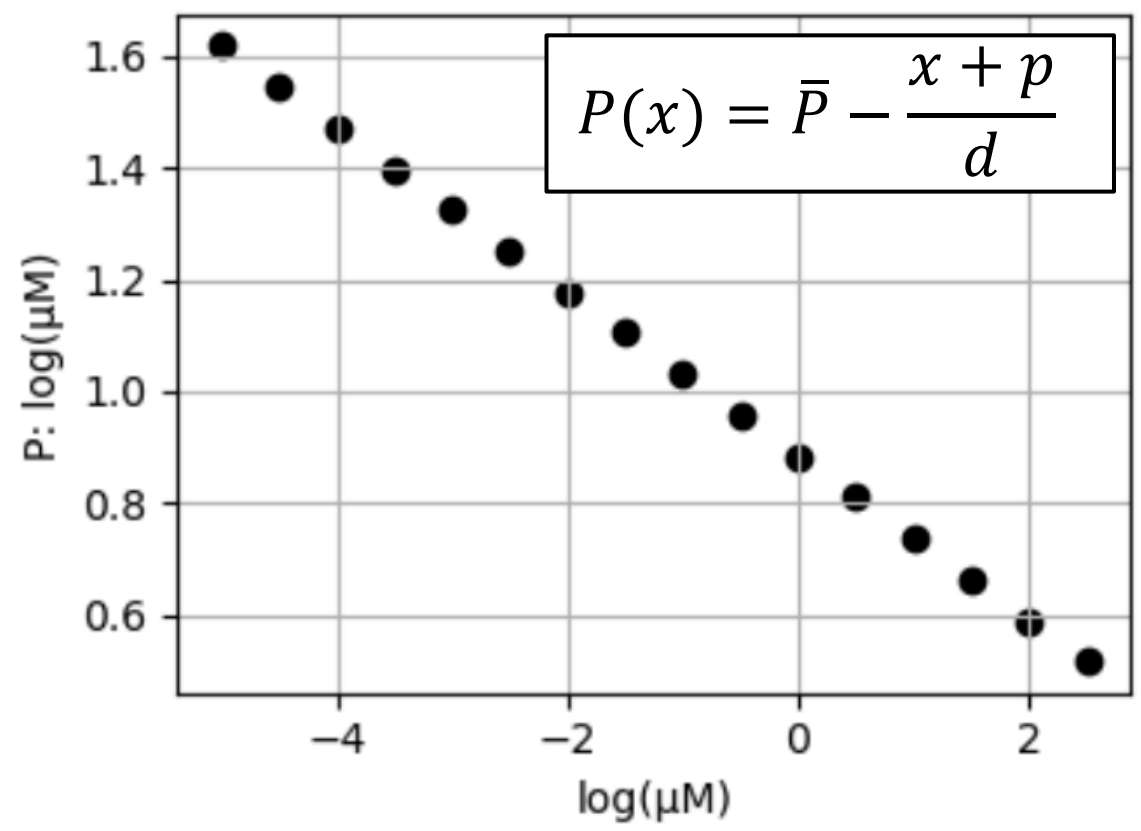

Supplementary Figure 5. Adaptative threshold for comparison of concentrations associated with special points on the fitted models. The threshold is required to exclude false positives where observed signal reduction is driven by cell death rather than receptor inhibition. We empirically calibrated the computational formula for the delay between activity and cytotoxicity signals. The separation threshold, P(x), with $\bar{P} = 1.618, d = 1.0638, p = -5$. Here, x represents the compound concentration associated with the observed activity in log(µM). The parameters $\bar{P}$ (reference separation desired at $x = p$ in log(µM)), p (positional shift), and the d (damping factor) was empirically optimized to align the computational output with expert assessment of the dose–response curves.

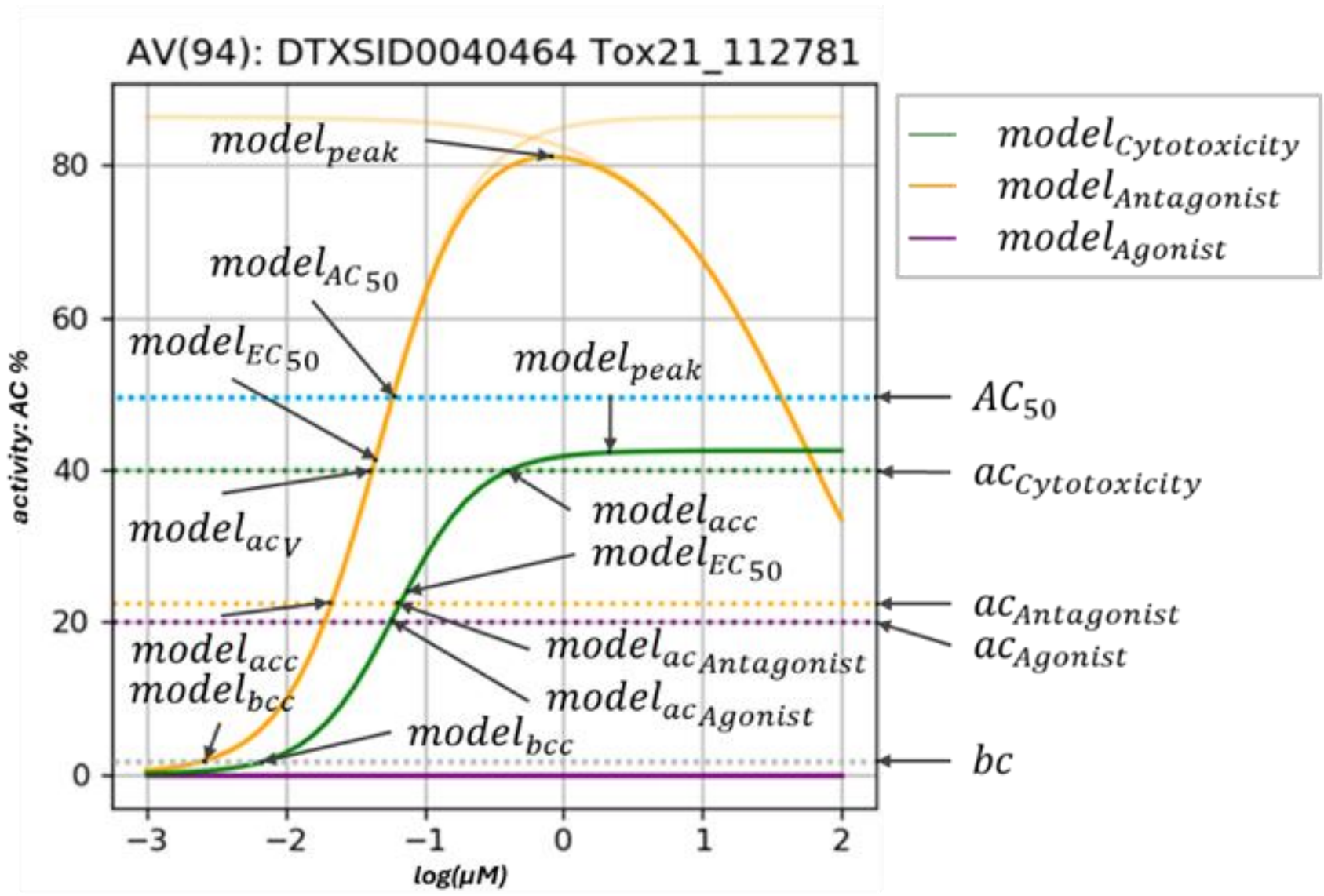

Supplementary Figure 6. Nomenclature of special points on each fitted model projected in the same dose-response space. Four points are considered for the comparison of an activity model ($model_{Activity} \equiv A$) (either agonist or antagonist activity) and a cytotoxicity model ($model_{Cytotoxicity} \equiv V$). The horizontal lines named "Viability cut-off" ($ac_{Cytotoxicity}$) and "activity cut-off" ($ac_{Activity}$) are the cut-offs defined by US EPA as the thresholds for positivity of the signal measured in the endpoint. The ratios between the concentrations of V_{acA} and acc_A and between acc_V and A_{acV} have been systematically computed. Hence, depending on additional specifics of the location and the shape of fitted models (detailed algorithm not disclosed) if both or one of the ratios is such that $AacV/accV < P(x)$ and $VacA/accA < P(x)$, then the positivity label ($True$) of the activity model was downgraded to "non-active" ($False$). Accordingly, when both ratios are greater than the corresponding $P(x)$, the two fitted models are considered significantly different and away from each other and thus the activity is not associated to an experimental artifact due to cytotoxicity. In such cases the positivity class of a given fitted model representative of an activity endpoint was preserved. Importantly, during our curation protocol positivity was never assigned to a fitted activity model. On the other hand, we set the positivity class for cytotoxicity if a fitted model of cytotoxicity signal served to downgrade a fitted model for activity.

Supplementary Table 1. Statistics of predictions for each molecular fingerprint of internal validation set.

Molecular fingerprint		Precision	Recall	F1-score	Support	C	Gamma	Kernel
Pubchem	0	0.996	1.000	0.998	1131	10	0.01	RBF
	1	1.000	0.925	0.961	53			
	Accuracy	0.997	0.997	0.997	-			
	Macro average	0.998	0.962	0.980	1184			
	Weighted average	0.997	0.997	0.997	1184			
Maccs	0	0.994	1.000	0.997	1131	10	0.01	RBF
	1	1.000	0.868	0.929	53			
	Accuracy	0.994	0.994	0.994	-			
	Macro average	0.997	0.934	0.963	1184			
	Weighted average	0.994	0.994	0.994	1184			
FCFP6	0	0.997	0.998	0.998	1131	10	0.001	RBF
	1	0.962	0.943	0.952	53			
	Accuracy	0.996	0.996	0.996	-			
	Macro average	0.979	0.971	0.975	1184			
	Weighted average	0.996	0.996	0.996	1184			

ECFP6	0	0.996	0.999	0.998	1131	10	0.01	RBF
	1	0.980	0.925	0.951	53			
	Accuracy	0.996	0.996	0.996	-			
	Macro average	0.988	0.962	0.975	1184			
	Weighted average	0.996	0.996	0.996	1184			

Supplementary Table 2. Confusion matrix for the model based on the training set.

		**Prediction**		
		**Active**	**Inactive**	**Total**
**Experimental**	**Active**	139	12	151
	**Inactive**	0	3193	3193
	**Total**	139	3205	3344

Supplementary Table 3. Confusion matrix for the model based on the external test set.

		**Prediction**		
		**Active**	**Inactive**	**Total**
**Experimental**	**Active**	71	10	81
	**Inactive**	2	1729	1731
	**Total**	73	1739	1812

Supplementary Table 4. Confusion matrix based on the substances of the external validation set falling inside model AD and associated with a reliable prediction.

		Prediction		
		Active	Inactive	Total
Experimental	Active	60	5	65
	Inactive	0	1452	1452
	Total	60	1457	1517

Supplementary Table 5. PubChem Assays IDs associated with datapoints retrieved from BindingDB for data augmentation of the modelling dataset with “Positive” entries

id	version	Name & reference: description
1797784	2	TRbeta-Binding Assay from Article 10.1021/jm021080f: "Thyroid receptor ligands. 1. Agonist ligands selective for the thyroid receptor beta1." // IC50 is the concentration of each compound required to inhibit 50% binding of 125I-T3 to hTRbeta.
1797782	2	TRalpha-Binding Assay. from Article 10.1021/jm021080f: "Thyroid receptor ligands. 1. Agonist ligands selective for the thyroid receptor beta1." // IC50 is the concentration of each compound required to inhibit 50% binding of 125I-T3 to hTRalpha.
1804101	3	transactivation assay from US Patent US10544075: "Derivatives of sobetirome" // Human epithelial kidney cells (HEK 293) were grown to 80% confluency in Dubelcco's modified Eagles 4.5 g/L glucose medium (high glucose DMEM) containing 10% fetal bovine serum, 50 units/mL penicillin and 50 μg/mL streptomycin. The cells were trypsinized with 0.25% trypsin, then diluted to 5×105 cells/mL with high glucose DMEM. Cells were added to Costar 3917 96-well plates at 5×104 cells/well, then incubated at 37° C. for 24 hours. 1.5 μg of TR expression vector (full length TRα-CMV or TRβ-CMV), 1.5 μg of a reporter plasmid containing a DR4 thyroid hormone response element (TRE) direct repeat spaced by four nucleotides (AGGTCAcaggAGGTCA) cloned upstream of a minimal thymidine kinase promoter linked to a firefly luciferase coding sequence, and 0.75 μg of a pRL-SV40 constitutive Renilla luciferase reporter plasmid were diluted into 540 μl of OptiMEM. 27 μL of lipofectamine reagent was diluted into 540 μL of OptiMEM. The plasmid and lipofectamine dilutions were combined then incubated at RT for 10 min. The mixture was then diluted into 4.29 mL of OptiMEM. Plates were washed with 100 μL of phosphate buffered saline (PBS) at pH 7.2 without magnesium or calcium chloride per well. Transfection mixtures were added at 50 μL per well, then incubated at 37° C. for 4 hours. Modified DME/F-12 Ham's medium without phenol red containing 15 mM HEPES and bicarbonate, 5 mM L-glutamine, charcoal-stripped FBS, 50 units/mL penicillin and 50 μg/mL streptomycin was added at 50 μL per well, then the plates were incubated at 37° C. for 20 hours. Drug stocks were made at 10 mM in DMSO, then serially diluted to 1× concentrations in DME/F-12 Ham's. Plates were washed with 100 μL of PBS (pH 7.2) per well. 100 μL of each drug stock was added to the wells in triplicate, and then the plates were incubated at 37° C. for 24 hours.Cells were assayed for luciferase activity using the Promega DualGlo kit. 50 μl of Luciferase Reagent were added per well, the plate was rocked for 15 min at RT, and then the plate was read for firefly luciferase activity. A 50 μl volume of Stop & Glo Reagent was added per well, then the plate

		was read for Renilla luciferase activity. Data normalized to Renilla internal control were analyzed with GraphPad Prism v.4a using the sigmoid dose response model to generate EC50 values±SEM.
1799414	3	Transactivation Assay in U2OS cells from Article 10.1021/cb600311v: "Design and characterization of a thyroid hormone receptor alpha (TRalpha)-specific agonist." // TRE-driven dual-luciferase reporter assay in human bone osteosarcoma epithelial (U2OS) cell line.
1799415	3	Transactivation Assay in HeLa cells from Article 10.1021/cb600311v: "Design and characterization of a thyroid hormone receptor alpha (TRalpha)-specific agonist." // TRE-driven dual-luciferase reporter assay in human uterine cervical cancer (Hela) cell line
1797786	2	TRalpha-Binding Assay and Thyroid Response Element (TRAFalpha1) Reporter Assay. from Article 10.1016/j.bmcl.2007.05.049: "Thyroid receptor ligands. Part 8: Thyromimetics derived from N-acylated-alpha-amino acid derivatives displaying modulated pharmacological selectivity compared with KB-141." // IC50 is the concentration of each compound required to inhibit 50% binding of 125I-T3 to hTRalpha. EC50 is the concentration of compound required to reach 50% of T3-induced TRalpha-activation in CHOK1 cells stably transfected with hTRalpha using alkaline phosphatase reporter assay.
1797795	2	TRalpha-Binding Assay. from Article 10.1016/j.bmcl.2005.11.002: "Thyroid receptor ligands. Part 4: 4'-amido bioisosteric ligands selective for the thyroid hormone receptor beta." // IC50 is the concentration of each compound required to inhibit 50% binding of 125I-T3 to hTRalpha.
1797797	2	TRalpha-Binding Assay. from Article 10.1016/j.bmcl.2004.04.032: "Thyroid receptor ligands. Part 2: Thyromimetics with improved selectivity for the thyroid hormone receptor beta." // IC50 is the concentration of each compound required to inhibit 50% binding of 125I-T3 to hTRalpha.

1797787	2	TRbeta-Binding Assay and Thyroid Response Element (TRAFbeta1) Reporter Assay. from Article 10.1016/j.bmcl.2007.05.049: "Thyroid receptor ligands. Part 8: Thyromimetics derived from N-acylated-alpha-amino acid derivatives displaying modulated pharmacological selectivity compared with KB-141." // IC50 is the concentration of each compound required to inhibit 50% binding of 125I-T3 to hTRbeta. EC50 is the concentration of compound required to reach 50% of T3-induced TRbeta-activation in CHOK1 cells stably transfected with hTRbeta using alkaline phosphatase reporter assay.
1797796	2	TRbeta-Binding Assay from Article 10.1016/j.bmcl.2005.11.002: "Thyroid receptor ligands. Part 4: 4'-amido bioisosteric ligands selective for the thyroid hormone receptor beta." // IC50 is the concentration of each compound required to inhibit 50% binding of 125I-T3 to hTRbeta.
1797789	2	TRalpha-Binding Assay and Thyroid Response Element (TRAFalpha1) Reporter Assay. from Article 10.1016/j.bmcl.2007.01.009: "Thyroid receptor ligands. Part 7: Indirect antagonists of the thyroid hormone receptor with improved affinity." // IC50 is the concentration of each compound required to inhibit 50% binding of 125I-T3 to hTRalpha. EC50 is the concentration of compound required to reach or inhibit (agonist or antagonist) 50% of T3-induced TRalpha-activation in CHOK1 cells stably transfected with hTRalpha using alkaline phosphatase reporter assay.
1797791	2	TRbeta-Binding Assay and Thyroid Response Element (TRAFbeta1) Reporter Assay. from Article 10.1016/j.bmcl.2007.01.009: "Thyroid receptor ligands. Part 7: Indirect antagonists of the thyroid hormone receptor with improved affinity." // IC50 is the concentration of each compound required to inhibit 50% binding of 125I-T3 to hTRbeta. EC50 is the concentration of compound required to reach or inhibit (agonist or antagonist) 50% of T3-induced TRbeta-activation in CHOK1 cells stably transfected with hTRbeta using alkaline phosphatase reporter assay.
1797785	2	TRbeta-Binding Assay and Thyroid Response Element (TRAFbeta1) Reporter Assay. from Article 10.1021/jm021080f: "Thyroid receptor ligands. 1. Agonist ligands selective for the thyroid receptor beta1." // IC50 is the concentration of each compound required to inhibit 50% binding of 125I-T3 to hTRbeta. EC50 is the concentration of compound required to reach 50% of T3-induced TRbeta-activation in CHOK1 cells stably transfected with hTRbeta using alkaline phosphatase reporter assay.
1797783	2	TRalpha-Binding Assay and Thyroid Response Element (TRAFalpha1) Reporter Assay. from Article 10.1021/jm021080f: "Thyroid receptor ligands. 1. Agonist ligands selective for the thyroid receptor beta1." // IC50 is the concentration of each compound required to inhibit 50% binding of 125I-T3 to hTRalpha. EC50 is the concentration of compound required to reach 50% of

		T3-induced TRalpha-activation in CHOK1 cells stably transfected with hTRalpha using alkaline phosphatase reporter assay.
1797790	2	TRbeta-Binding Assay from Article 10.1016/j.bmcl.2007.01.009: "Thyroid receptor ligands. Part 7: Indirect antagonists of the thyroid hormone receptor with improved affinity." // IC50 is the concentration of each compound required to inhibit 50% binding of 125I-T3 to hTRbeta.
1797805	2	TRbeta-Binding Assay from Article 10.1021/jm060521i: "Thyroid receptor ligands. 6. A high affinity "direct antagonist" selective for the thyroid hormone receptor." // IC50 is the concentration of each compound required to inhibit 50% binding of 125I-T3 to hTRbeta.
1797803	2	TRalpha-Binding Assay. from Article 10.1021/jm060521i: "Thyroid receptor ligands. 6. A high affinity "direct antagonist" selective for the thyroid hormone receptor." // IC50 is the concentration of each compound required to inhibit 50% binding of 125I-T3 to hTRalpha.
1797788	2	TRalpha-Binding Assay. from Article 10.1016/j.bmcl.2007.01.009: "Thyroid receptor ligands. Part 7: Indirect antagonists of the thyroid hormone receptor with improved affinity." // IC50 is the concentration of each compound required to inhibit 50% binding of 125I-T3 to hTRalpha.
1797792	2	TRalpha-Binding Assay and Thyroid Response Element (TRAFalpha1) Reporter Assay. from Article 10.1021/jm050004k: "Thyroid receptor ligands. 3. Design and synthesis of 3,5-dihalo-4-alkoxyphenylalkanoic acids as indirect antagonists of the thyroid hormone receptor." // IIC50 is the concentration of each compound required to inhibit 50% binding of 125I-T3 to hTRalpha. EC50 is the concentration of compound required to inhibit 50% of T3-induced TRalpha-activation in CHOK1 cells stably transfected with hTRalpha using alkaline phosphatase reporter assay.
1797793	2	TRbeta-Binding Assay and Thyroid Response Element (TRAFbeta1) Reporter Assay. from Article 10.1021/jm050004k: "Thyroid receptor ligands. 3. Design and synthesis of 3,5-dihalo-4-alkoxyphenylalkanoic acids as indirect antagonists of the thyroid hormone receptor." // IC50 is the concentration of each compound required to inhibit 50% binding of 125I-T3 to hTRbeta. EC50 is the concentration of compound required to inhibit 50% of T3-induced TRbeta-activation in CHOK1 cells stably transfected with hTRbeta using alkaline phosphatase reporter assay.
1797804	2	TRalpha-Binding Assay and Thyroid Response Element (TRAFalpha1) Reporter Assay. from Article 10.1021/jm060521i: "Thyroid receptor ligands. 6. A high affinity "direct antagonist" selective for the thyroid hormone receptor." // IC50 is the concentration of each compound required to inhibit 50% binding

		of 125I-T3 to hTRalpha. EC50 is the concentration of compound required to inhibit 50% of T3-induced TRalpha-activation in CHOK1 cells stably transfected with hTRalpha using alkaline phosphatase reporter assay.
1797806	2	TRbeta-Binding Assay and Thyroid Response Element (TRAFbeta1) Reporter Assay. from Article 10.1021/jm060521i: "Thyroid receptor ligands. 6. A high affinity "direct antagonist" selective for the thyroid hormone receptor." // IC50 is the concentration of each compound required to inhibit 50% binding of 125I-T3 to hTRbeta. EC50 is the concentration of compound required to inhibit 50% of T3-induced TRbeta-activation in CHOK1 cells stably transfected with hTRbeta using alkaline phosphatase reporter assay.
1804893	3	Time-Resolved Fluorescence Resonance Energy Transfer (TR-FRET) Assay for Thyroid Hormone Receptor Agonist Screening from US Patent US10800767: "Thyroid hormone receptor beta agonist compounds" // LanthaScreen TR-FRET Thyroid Receptor alpha Coactivator Assay kit (ThermoFisher) and LanthaScreen TR-FRET Thyroid Receptor beta Coactivator Assay kit (ThermoFisher) were used for agonist compound screening. Compounds in DMSO were diluted using ECHO Liquid Handler (Labcyte Inc.) into 384 plates in 10-point 3-fold series in duplicate (5 micro M final top concentration). Buffer C (ThermoFisher) was added to each well before the 4× mixture of fluorescein-SCR2-2 coactivator (200 nM final concentration), Terbium-labeled anti-GST antibody (2 nM final concentration), and TR alpha-LBD (0.4 nM final concentration) or TR beta-LBD (1.0 nM final concentration) was added. After 2 hour incubation at room temperature in dark, the TR-FRET signal was measured on an EnVision plate reader (PerkinElmer) with excitation at 340 nm and dual emission readout at 495 and 520 nm with the delay time of 100 micro second and the integration time of 200 micro second. The ratio of emission signal at 520 and at 495 was used to calculate EC50 using GraphPad Prism (GraphPad Software). In every batch of compound screening, T3 (L-3,3′,5-Triiodothyronine sodium salt, >95%) (Calbiochem) was used as reference compound. The EC50 of T3 measured were within 3-fold of the reference value provided by the assay kit manufacturer (ThermoFisher Scientific). The Z′ factors measured in every batch of screening using T3 as high percent effect (HPE) control and 0.5% DMSO as zero percent effect (ZPE) control were in the range of 0.5 to 0.8. Compounds' THR-beta selectivity values are derived from T3-selectivity normalized data.

Supplementary Table 6. Counts of entries common or different between the latest invitrodb-v-4- 2 (US EPA., 2024.) vs. the KREATiS automated curation invitrodb-v-3-3 (US EPA., 2021)

Endpoint	Entries / Class label	A: invitrodb-v-4-2	$A \cap B$	B: KREATiS curated (invitrodb-v.3.3)
Agonist	Entries	2197	6108	0
	Positive	4	19	2
	Negative	2	6083	4
Antagonist	Entries	2197	6108	0
	Positive	1354	98	9
	Negative	9	4647	1354
Viability	Entries	2197	6108	0
	Positive	0	920	442
	Negative	442	4746	0

Supplementary Table 7. Percentiles of threshold crossing concentrations (log(µM)) across all endpoints for the not curated dataset

$percentile$	$acc_{Agonist}$	$acc_{Antagonist}$	$acc_{Viability}$
0	-6.2587	-5.6319	-5.1208
10	-2.6078	0.0957	0.4860
20	-0.6809	0.7051	0.9437
30	0.6375	1.0707	1.1725
40	1.2460	1.1925	1.4014
50	1.6516	1.4363	1.5158
60	2.0573	1.5581	1.6302
70	2.0573	1.6800	1.7446
80	2.0573	1.8019	1.8591
90	2.0573	1.9237	2.0879
100	3.8828	6.5546	6.3216

Supplementary Table 8. Distribution parameters for threshold crossing concentrations (*log(μM)*) across all endpoints for the not curated dataset

Endpoint	Mean	std	median
Agonist	0.6681	1.9577	1.6276
Antagonist	1.1151	1.0064	1.3690
Viability	1.2843	0.8598	1.4978

Supplementary Table 9. Percentiles for the differences in threshold crossing concentrations (*log(µM)*) across activity endpoints vs. cytotoxicity for the not curated dataset

$percentile$	$acc_{Antagonist} - acc_{Viability}$	$acc_{Aagonist} - acc_{Viability}$
0	-6.6078	-6.3744
10	-0.7263	-3.5462
20	-0.5043	-2.1321
30	-0.3933	-1.5882
40	-0.2824	-1.0443
50	-0.1714	-0.7180
60	-0.0604	-0.5004
70	-0.0604	-0.3916
80	0.0505	-0.0653
90	0.0505	0.1523
100	4.4895	4.5033

Supplementary Table 10. Distribution parameters for the differences in threshold crossing concentrations (*log(μM)*) across activity endpoints vs. cytotoxicity for the not curated dataset

$\mathrm{acc}_{\mathrm{Activity}} - \mathrm{acc}_{\mathrm{Viability}}$	mean	std	median
Agonist	-1.1728	1.6032	-0.7781
Antagonist	-0.3329	0.5673	-0.2217
